# Dissecting the subcellular membrane proteome reveals enrichment of H^+^ (co-)transporters and vesicle trafficking proteins in acidic zones of *Chara* internodal cells

**DOI:** 10.1371/journal.pone.0201480

**Published:** 2018-08-29

**Authors:** Heidi Pertl-Obermeyer, Peter Lackner, Waltraud X. Schulze, Marion C. Hoepflinger, Margit Hoeftberger, Ilse Foissner, Gerhard Obermeyer

**Affiliations:** 1 Molecular Plant Biophysics and Biochemistry, Department of Biosciences, University of Salzburg, Salzburg, Austria; 2 Bioinformatics of Allergens, Department of Biosciences, University of Salzburg, Salzburg, Austria; 3 Plant Systems Biology, University of Hohenheim, Stuttgart, Germany; 4 Plant Cell Dynamics, Department of Biosciences, University of Salzburg, Salzburg, Austria; The University of Melbourne, AUSTRALIA

## Abstract

The Characeae are multicellular green algae with very close relationship to land plants. Their internodal cells have been the subject of numerous (electro-)physiological studies. When exposed to light, internodal cells display alternating bands of low and high pH along their surface in order to facilitate carbon uptake required for photosynthesis. Here we investigated for the first time the subcellular membrane protein composition of acidic and alkaline regions in internodal cells of *Chara australis* R. Br. using MS-proteomics. The identified peptides were annotated to *Chara* unigenes using a custom-made *Chara* database generated from a transcriptome analysis and to orthologous *Arabidopsis* genes using TAIR (The Arabidopsis Information Resource) database. Apart from providing the first public-available, functionally-annotated sequence database for *Chara australis*, the proteome study, which is supported by immunodetection, identified several membrane proteins associated with acidic regions that contain a high density of specific plasma membrane (PM) invaginations, the charasomes, which locally increase the membrane area to overcome diffusion limitation in membrane transport. An increased abundance of PM H^+^ ATPases at charasomes is consistent with their role in the acidification of the environment, but the characean PM H^+^ ATPase sequence suggests a different regulation compared to higher plant PM H^+^ ATPases. A higher abundance of H^+^ co-transporters in the charasome-rich, acidic regions possibly reflects enhanced uptake of ions and nutrients. The increase in mitochondrial proteins confirms earlier findings about the accumulation of cortical mitochondria in the acidic zones. The significant enrichment of clathrin heavy chains and clathrin adaptor proteins as well as other proteins involved in trafficking indicate a higher activity of membrane transport in the charasome-rich than in charasome-poor areas. New and unexpected data, for instance the upregulation and abundance of vacuolar transporters correlating with the charasome-rich, acidic cell regions account for new perspectives in the formation of charasomes.

## Introduction

Since more than 6 decades, characean internodal cells have been used to study transport processes and to characterize transporter proteins by means of (electro-) physiological techniques [1, 2], microscopy [3] and biochemistry including proteomics [4] and metabolomics [5]. Characean internodal cells have a cylindrical shape and are up to several centimetres long. Their cytoplasm consists of a stationary layer of helically arranged chloroplasts, the streaming endoplasm and a huge vacuole (see S1 Fig and [6, 7]) for overview). Just as higher aquatic plants, the characean algae face the problem that in their aqueous environment, carbon, required for photosynthesis, is mainly present as HCO_3_^-^ (bicarbonate or hydrogen carbonate). Contrary to CO_2_, which is the main carbon source for land plants, HCO_3_^-^ is poorly membrane permeable. The plasma membrane is thus equipped with numerous H^+^ ATPases, which acidify the external environment and convert HCO_3_^-^ into easily permeable CO_2_, either directly or indirectly via carbonic anhydrase [8–12]. Characean internodal cells produce extended acidic regions (H^+^ efflux) along their surface with alternating smaller alkaline areas with probably local OH^-^ efflux [2], required to achieve pH homoeostasis of the cytoplasm. This pH banding pattern can be visualized by phenol red [13] and acidic bands correlate with an enhanced photosynthesis compared to alkaline regions [14, 15] as well as high abundance and activity of cortical mitochondria [3] (S1 Fig).

Quite early, specialized plasma membrane invaginations, so-called charasomes, were discovered ([16, 17], see also S1 Fig)). Under steady-state conditions, the pH banding correlates with the abundance of charasomes in internodal cells of the genus *Chara* [3, 18]. Charasomes are convoluted plasma membrane domains, which can be visualized in living cells with fluorescent plasma membrane dyes due to the increased signal caused by the superimposed plasma membrane invaginations [3]. They are up to several μm large and abundant in acidic regions but absent or fewer and smaller in alkaline regions. It is assumed that this correlation between pH banding pattern and charasome occurrence is caused by the high number of H^+^ ATPases accommodated in charasomes [3, 8]. The pH banding pattern also correlates with the differential distribution of cortical mitochondria. Similar to charasomes, they are longer and more abundant at acidic bands but smaller and less frequent in alkaline regions [3]. Additional functions for charasomes, like chloride transport, have also been proposed [19–21]. Charasome formation is reversible, allowing adaptation to different light conditions.

Charasomes are degraded upon dark incubation and they re-appear when cells are exposed to light [22–24]. Recently it was shown that charasomes are formed via exocytosis of trans Golgi network-derived material and local inhibition of endocytosis (coated vesicle detachment) that probably involves a phosphoinositide 4 kinase [25], whereas degradation of charasomes occurs via clathrin-dependent membrane retrieval [22].

Since their discovery these charasome membranes were thought to have an important role in the localized transport of nutrients, but despite intensive investigation of their function, almost no molecular data, e.g. proteins functionally active in the charasome membrane, are available. Instead of studying individual putative candidates involved in the function of charasomes, a comprehensive insight in the physiological function of charasomes and their putative contribution to cellular processes can be obtained by analysing the subcellular membrane proteome revealing the molecular composition of charasomes. Hence, a first proteomics study of the *Chara* membranes was performed together with RNAseq analysis to provide a *Chara*-specific database suitable for the functional annotation of the identified peptides. Together, charophyte green algae and the embryophytes form the clade Streptophyta [26–28] indicating the close relationship of the Characeae with higher plants. Hence, peptides identified by MS were not only annotated to *Chara* unigenes but also to orthologous *Arabidopsis* genes. In contrast to recent transcriptome analyses of *Chara vulgaris* [26, 29], *Chara globularis* [30] and *Nitella mirabilis* [31] that identified a large number of transcript sequences to study the evolution of Characeae in relation to other algae families and higher plants, the present study is driven by the question of membrane proteins and their physiological role of charasomes.

Therefore, we correlated the identified membrane proteins with the presence of charasomes and the occurrence of acidic and alkaline regions along single *Chara* cells. The results reflect the putative function of charasomes in transport of ions and nutrients, thus promoting carbohydrate and energy metabolism in the charasome-rich regions, and support earlier observations about their formation and degradation. New and unexpected results provide the basis for further studies. Considering the fact that amplification of membrane area is a widespread strategy to overcome diffusion limitations, our data are of general relevance for membrane biology.

## Materials and methods

### Plant material and culture conditions

For this study, we investigated male thalli of *Chara australis* R. Br. In our earlier articles, we referred to this alga as *C*. *corallina* according to the monograph of Characeae by Wood and Imahori [32] who amalgamated both species. The fact that *C*. *australis* is dioecious and possesses only the half number of chromosomes (14) compared with the monoecious *C*. *corallina* (≥28), however, justifies the retainment of a separate species [33]. The alga used in this study is dioecious and the number of DAPI-stained chromosomes in developing antheridia is 14; it was therefore classified as *C*. *australis*.

Thalli were grown at 20°C in 10–50 l aquaria filled with distilled water and a substrate of soil, peat and sand. Fluorescent lamps provided a 14/10 h light/dark cycle and light intensity was low (about 5 μE m^−2^ s^−1^ at the water surface) in order to prevent calcification and extensive growth of epiphytes. *C*. *australis* thalli used for this study were ca. 8 weeks old and collected 6 h after the end of a regular night.

### Confocal microscopy

The confocal laser scanning microscopy was performed with a Leica TCS SP5 (Mannheim, Germany) coupled to a DMI 6000B inverted microscope or a Zeiss (Jena, Germany) LSM 510 coupled to a Zeiss Axiovert inverted microscope. Fluorescent dyes and instrument settings were as described in Schmoelzer et al. [3].

### RNAseq analysis

Thalli were collected and rinsed with distilled water, gently blotted dry, immediately frozen in liquid nitrogen, and homogenized to a fine powder using mortar and pestle. RNA was isolated using a slightly modified protocol described by Sangha et al. [34]. In brief, about 0.5 g fresh weight of frozen thalli powder was transferred to a pre-chilled 50 ml centrifugation tube and solved in 5 ml of preheated (65°C) total RNA extraction buffer (2% (w/v) cetyltrimethylammonium bromide, 1% (w/v) polyvinylpyrrolidone, 20 mM ethylenediaminetetraacetic acid (EDTA), 1.4 M NaCl, 2% β-mercaptoethanol, and 100 mM tris(hydroxymethyl)aminomethane (Tris) adjusted to pH 8.0 with HCl). Samples were incubated for 30 min at 65°C in a water bath. During incubation, the samples were vortexed every 5 min. Then, an equal volume of chloroform:isoamylalcohol (24:1) was added, the samples were mixed by vortexing for 30 s and centrifuged at 10,000 x*g* for 20 min at 4°C. The aqueous supernatant (about 4 ml per tube) was carefully transferred into four 2 ml RNase-free microcentrifuge tubes (ca. 1 ml supernatant each). Again, an equal volume of chloroform:isoamylalcohol mixture was added, vortexed and centrifuged at 10,000 x*g* for 10 min at 4°C. The supernatants (about 0.8 ml/tube) were transferred to RNase-free 1.5 ml microcentrifuge tubes and 0.4 ml of 96–100% ethanol was added. All four samples of one extraction were pooled, immediately loaded onto an RNA-binding column (0.75 ml per column, RNeasy Plant Mini kit, Qiagen, Vienna, Austria) and spun at 10,000 x*g* for 30 s at room temperature (RT). Once samples were completely loaded, columns were washed and desalted following the manufacturer’s protocol. Finally, RNA was eluted using 50 μl of RNase-free water. RNA quality and integrity were checked using a spectrophotometer (NanoDrop, Thermo Scientific) as well as agarose gel electrophoresis. Pooled RNA extractions were send to BGI Genomics (Hongkong, China) for cDNA generation and *de novo* sequencing of the *Chara australis* transcriptome (single replicate) using the Illumina Hiseq 2000 technology [22, 35]. Raw sequence data were filtered and the resulting clean reads were assembled to unigenes using Trinity software [36]). Finally, blastx alignment between unigenes and protein databases (NCBI non-redundant (NR), Swiss-Prot, KEGG and COG) was performed. The best aligning result was used to decide the sequence direction of each unigene. In a next step, the possible functions were annotated to the identified unigene sequences using BLAST software and the following databases: NT (release 20130408), NR (release 20130408), KEGG (release 63.0), Swiss-Prot (release-2013_03) and COG (release 20090331). In addition, unigenes were also classified to MapMan BINs (functional classes) using the Mercator pipeline (www.plabipd.de) searching the databases TAIR (release 10), PPAP (Swiss-Prot Plant Protein), JGI Chlamy (release 4), *Physcomitrella* and *Selaginella* database as well as enabling InterProScan. RNAseq replicates were n = 1. RNAseq data were submitted to EBI-ENA data base (accession no. ERP023711). The sequence of the *C**hara* *a**ustralis* PM H^+^ ATPase CaHA1 was deposited at NCBI (access. no. MF196972).

### Preparation of organelle membrane fractions

Membrane fractions (MF) of *Chara australis* internodal cells were prepared by differential centrifugation [37]. After determination of fresh weights (3 to 9.5 g), *Chara* cells were frozen in liquid nitrogen, transferred into pre-cooled mortars and ground with the pistil to small pieces. Cell pieces were resuspended in ice-cold homogenization buffer (330 mM sucrose, 100 mM KCl, 1 mM EDTA, 50 mM Tris adjusted with 2-(N-morpholino)ethanesulfonic acid (MES) to pH 7.5, 5 mM dithiotreitol (DTT)) containing a protease inhibitor cocktail (10 μM leupeptin, 1 μM pepstatin A, 1 mM phenylmethylsulfonyl fluoride (PMSF), 2 μM E-64) and were further homogenized with a Teflon Potter-Elvehjem-type homogenizer on ice. The homogenate was filtered through a 21 μm nylon mesh, and centrifuged at 7,500 x*g* for 15 min at 4°C. Finally, the supernatant was centrifuged at 48,000 *xg* for 80 min at 4°C. The resulting pellet, the membrane fraction (MF) was stored at -80°C.

To isolate alkaline and acid regions from *Chara* internodal cells, alkaline bands were visualized with 1 μM phenol red dissolved water and separated by cutting the internodes followed by rapid freezing of the cell pieces in liquid nitrogen. The collected fragments retained their cortical organelles, and the charasomes at the acid regions remained in place although most of the endoplasm and the vacuole were lost (see Results). MFs from the collected alkaline as well as from the acid bands were prepared as described above.

### Gel electrophoresis and immunodetection

Membrane fractions (MFs, 10–30 μg) were denatured in sample buffer at 56°C for 10 min, loaded onto a discontinuous gel system with 4% stacking gel and 10 or 12.75% separation gel (Protean 3 system, Bio-Rad, Vienna, Austria). Proteins were stained with Coomassie Brilliant Blue R-250 (CBB).

For immunodetection, separated proteins were transferred onto PVDF membranes (Roth, Karlsruhe, Germany) by electro-transfer with 20 V for 1 h (Semi Dry Electrophoretic Transfer Cell, Bio-Rad, Vienna, Austria). For detection of membrane and membrane-associated proteins, the following antibody dilutions and combinations were used: anti-PM H^+^-ATPase (1:1,000, Agrisera AS07 260, Vännäs, Sweden) and goat anti-rabbit IgG AP-conjugated or monoclonal anti-rabbit IgG AP-conjugated (1:5,000, Sigma, Vienna, Austria), anti-VHA-ɛ (1:2,000, Agrisera AS07 213) and anti-H^+^ PPase (1:2,000, gift from Prof. Maeshima, Nagoya University, Japan) with goat anti-rabbit IgG AP-conjugated (1:5,000 or 1:8,000, Sigma), anti-BiP2 (1:2,000, Agrisera AS09 466) and monoclonal anti-rabbit IgG AP-conjugated (1:5,000, Sigma), anti-tyrosine tubulin (1:800, Sigma) and goat anti-mouse IgG AP-conjugated (1:5,000, Sigma), anti-ARA6 (1:200, [38]) and goat anti-mouse IgG AP-conjugated (1:5,000, Sigma), anti-GRF (1:2,000, Agrisera AS12 2119) and goat anti-rabbit IgG AP-conjugated (1:10,000, Sigma) for detection of 14-3-3 proteins.

### ATP hydrolysis assay

Membrane fractions were tested for vanadate-sensitive and fusicoccin (FC)-stimulated ATP hydrolysis activity according to Pertl et al. [39]. Briefly, 10 μg protein of MF vesicles were incubated for 1 h at room temperature in ATPase reaction buffer containing 25 mM K_2_SO_4_, 50 mM MES/ Tris, pH 6.8, 0.1 mM EDTA, 4 mM MgSO_4_, 100 μM sodium molybdate, 0.005% Triton X-100, 1 mM NaN_3_, ± 250 μM vanadate. The reaction was started by addition of 3 mM ATP. After 1 h, 700 μl of the combined stopping and colour reagent was added [40]. The reagent was prepared freshly by mixing 6 parts 0.42% (w/v) ammonium molybdate, 28.6 ml H_2_SO_4_, 2% (w/v) SDS with one part 10% (w/v) ascorbic acid. After incubation at room temperature for 1 h, the absorbance was measured at 820 nm and the corresponding Pi concentrations were calculated from a calibration curve. All data were measured in triplicates from 3 independent preparations.

### Mass spectrometry

Membrane fraction proteins (5 μg) were denatured in UTU buffer (6 M urea, 2 M thiourea, pH 8.0). After reduction with 6.5 M DTT and alkylation of cysteine residues in 27 mM iodoacetamide, proteins were digested for 3 h by LysC (Wako Chemicals, Neuss, Germany) at RT. The solution was then diluted fourfold with 10 mM Tris-HCl, pH 8.0 followed by overnight digestion with trypsin (sequencing grade, Promega, Mannheim, Germany) at 37°C with shaking at 350 rpm. Digested peptides were desalted and concentrated over C18 STAGE-tips, dried in a vacuum concentrator and stored at -80°C.

For mass spectrometric analysis samples were suspended in resuspension buffer (0.2% v/v TFA, 5% v/v acetonitrile) and analysed by LC/MS/MS using nanoflow Easy-nLC1000 (Thermo Scientific, Darmstadt, Germany) as an HPLC-system and a Quadrupole-Orbitrap hybrid mass spectrometer (Q-Exactive Plus, Thermo Scientific) as a mass analyser. Peptides were eluted from a 75 μm x 50 cm C18 analytical column (PepMan, Thermo Scientific) on a linear gradient from 4 to 64% acetonitrile in 120 min and sprayed directly into the Q-Exactive mass spectrometer. Proteins were identified by MS/MS using information-dependent acquisition of fragmentation spectra of multiple charged peptides. Up to twelve data-dependent MS/MS spectra were acquired for each full-scan spectrum acquired at 70,000 full-width half-maximum resolution. Fragment spectra were acquired at a resolution of 35,000. Overall cycle time was approximately one second.

### Proteome analysis

Protein identification and ion intensity quantitation was carried out with MaxQuant software version 1.5.3.8 [41]. Spectra were matched against a custom-made *Chara* database or the *Arabidopsis* proteome (TAIR10, 35,386 entries) using Andromeda [42]. Thereby, carbamidomethylation of cysteine was set as a fixed modification; oxidation of methionine was set as variable modification. Mass tolerance for the database search was set to 20 ppm on full scans and 0.5 Da for fragment ions. Multiplicity was set to 1. For label-free quantitation, retention time matching between runs was chosen within a time window of two minutes. Peptide false discovery rate (FDR) and protein FDR were set to 0.01, while site FDR was set to 0.05. Hits to contaminants (e.g. keratins) and reverse hits identified by MaxQuant were excluded from further analysis.

To obtain the *Chara* protein database, all assembled unigenes were translated into 6 frames of amino acid sequences with the longest ORF selected for the database in FASTA format (S5 Table). For (semi-)quantitative analysis the data from the MaxQuant output (evidence.txt) were merged with the data of the Mercator analysis resulting in S3 Table, which lists the identified peptides found for each unigene annotated to orthologous *Arabibidopsis* genes. Only peptides found in all biological replicates were listed. The fraction of total peptide numbers was determined for each BIN classes.

In addition, the reported peptide ion intensity values were used for label-free data analysis by cRacker [43]. All proteotypic peptides were used for quantitation. Within each sample, ion intensities of each peptide ions species (each m/z) were normalized against the total ion intensities in that sample (peptide ion intensity/total sum of ion intensities). Subsequently, each peptide ion species (i.e. each m/z value) was scaled against the average normalized intensities of that ion across all treatments. For each peptide, values from at least three biological replicates then were averaged after normalization and scaling protein ion intensity sums were calculated from normalized scans scaled ion intensities of all proteotypic peptides. The mass spectrometry proteomics data have been deposited to the ProteomeXchange Consortium via the PRIDE [44] partner repository with the dataset identifier PXD006785.

## Results

### Transcriptome of *Chara* australis

**Fig 1:**
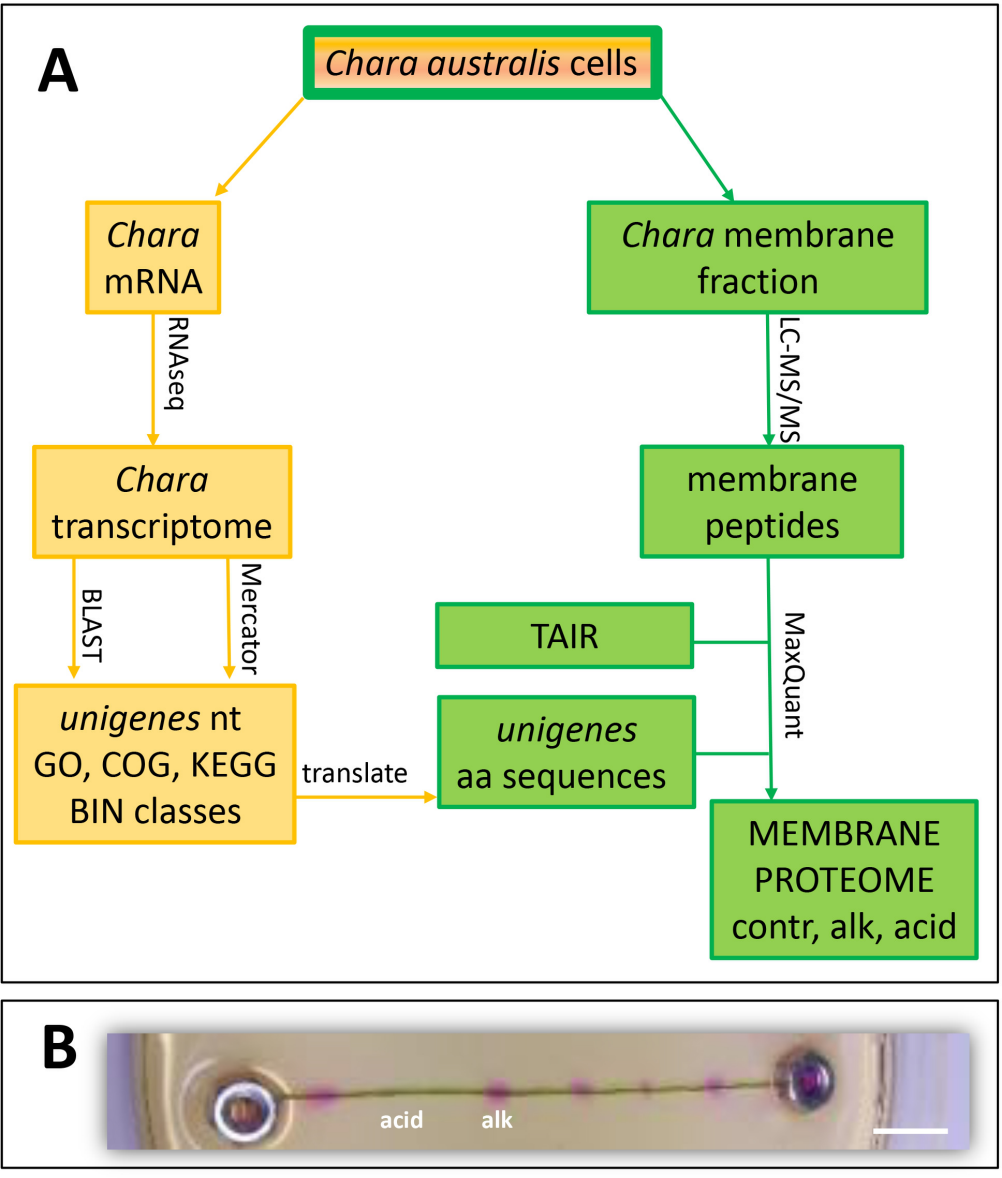
Summary of experimental set-up. (A) Membrane proteins and mRNA were isolated from *Chara australis* internodal cells incubated at ambient day/night cycles with 14 h light (contr). mRNA was subjected to RNAseq analysis and the assembled unigenes (nt, nucleotide sequence) were annotated to functional classes with BLAST using the databases GO, COG, KEGG and NR. Additionally, Mercator software classified the unigenes into functional categories (BIN classes), by combining BLAST searches and InterProScan using the databases TAIR, Uni/SwissProt, COG, cdd and InterProScan. Unigenes were also translated into amino acid sequences (aa), thus serving as a protein database in addition to TAIR for the membrane proteome analysis with MaxQuant software. See Material & Methods section for abbreviations of databases. (B) Acidic (acid) and alkaline (alk) areas of single internodal *Chara* cells were separated and collected for membrane preparation. Alkaline areas were visualized with phenol red. To facilitate collection, internodal cells were fixed by the weight of stainless steel nuts. Bar = 5 mm.

A comprehensive view of the distribution of membrane and membrane-associated proteins between charasome-enriched regions of *Chara* cells helps to understand the physiological function of these specific plasma membrane regions in more detail. Because sequence information of the *Chara* species is still scarce, e.g. *Chara* sequences in public databases show 1,231 nucleotide or 1,755 protein entries in NCBI in January 2018, an RNAseq analysis was performed prior to the proteome analysis to obtain sufficient sequence information to generate a custom-made *Chara* database for the proteome analysis. The experimental strategy is summarized in Fig 1 showing the RNAseq part in yellow and the proteome part in green. De novo transcriptome sequencing of a cDNA library prepared from *Chara* thalli was performed by BGI (Hong Kong, China) using the Illumina Hiseq 2000 platform resulting in clean reads of 4.788 Gb total nucleotides (nts) that could be assembled to 49,842,575 unigenes with a mean length of 619 bp (S1 Table). The number of unigenes decreased with unigene length (S2 Fig): approximately 2,000 unigenes had a length of 1,000 nt and ca. 100 unigenes had a length of 3,000 nt which likely correspond to putative full-length open reading frames (ORFs) suitable for the proteome analysis. In total 35,188 of all unigenes could be annotated to specific functions or functional classes (S2 Table). Most unigenes showed highest similarities to sequences of *Physcomitrella patens* (16.46%), followed by *Selaginalla moellendorffii* (8.29%), *Volvox carteri* (3.97%), *Picea sitchensis* (3.83%) and *Hordeum sativum* (3.58%) when compared to annotated sequences of the NCBI non-redundant (NR) database (S3 Fig). The fact that only 1.62% of all unigene sequences could be assigned to *Chara vulgaris* is probably due to the extremely scarce information on *Chara* sequences (664 sequences in NCBI NR, Jan 2018) that is typically for non-genome-sequenced organisms. When the *Chara* unigenes were classified into COG (Clusters of Orthologous Groups of proteins) or GO (Gene ontology) categories (S4 and S5 Figs), a first impression on the physiological status was gained. Most unigenes belonged to the classes ‘Translation, ribosomal structure and biogenesis’ and ‘Transcription’ as well as ‘Replication, recombination and repair’ followed by ‘Carbohydrate transport and metabolism’, ‘Posttranslational modification, protein turnover, chaperones’ and ‘Cell wall/membrane biogenesis’ (S4 Fig). Approximately 750 and 1,200 unigenes could be classified in the categories ‘Inorganic ion transport and metabolism’ and ‘Intracellular trafficking, secretion and vesicular transport’, respectively, which were suggested to be important for charasome function and development (COG, S4 Fig). Almost similar classifications were observed when unigenes were categorised into MapMan BINs using Mercator software, which classified 788 unigenes to ‘transport’ and 1,001 to ‘cell’ including vesicle transport and cell organisation (S6 Fig).

### PM H^+^ ATPase, vacuolar proton pumps and organelle marker proteins

To check the suitability of the *Chara* transcriptome for serving as a database for proteome analysis, the transcriptome was searched for P-type H^+^ ATPase sequences because the plasma membrane H^+^ ATPase is a major component of the charasomes in the acidic region (S1 Fig; [3]). Eight unigenes revealed high sequence similarities with the *Arabidopsis* H^+^ ATPase AHA2 with 1 unigene (CL2034.c1) identical to a previous identified *Chara australis* PM H^+^ ATPase CaHA1 (access. no. MF196972) with 985 amino acids and a predicted molecular weight of 107.6 kDa. An alignment of unigene amino acid sequences with AHA2 and CaHA1 is shown in S7 Fig, also indicating predicted transmembrane domains. Peptides identified by mass spectrometry analysis were marked and indicate at least two PM H^+^ ATPases present in the plasma membrane of *Chara* because unigene sequences differed in many sections of the PM H^+^ ATPase sequence.

**Fig 2:**
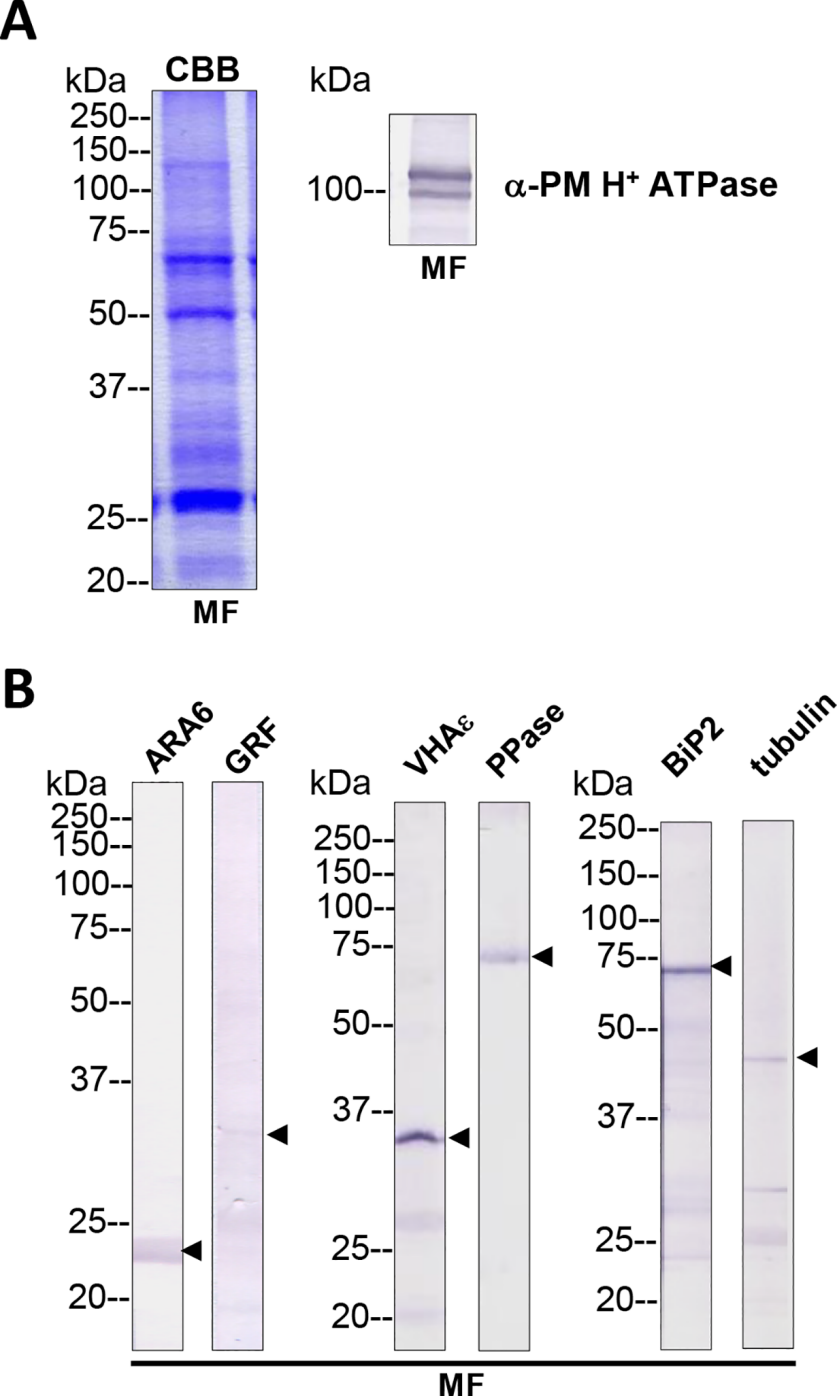
Total membrane fractions of *Chara* internodal cells. (A) Proteins of membrane fractions (MF) were separated by SDS-PAGE (10%), stained with Coomassie Brilliant Blue (CBB) or blotted onto PVDF membranes for immunodetection of the plasma membrane H^+^ ATPase. 30 μg protein per lane. Numbers on the left refer to molecular weight markers in kDa. Only the upper part of the PVDF membrane was used, the lower part was probed for immunodetection of low molecular weight proteins. (B) Immunodetection of selected organelle marker proteins for vacuoles (VHA-ɛ, H^+^ PPase), ER (BiP2), plasma membrane and endosomal compartments (ARA6) or cytosol (tubulin, GRF 14-3-3). Proteins of the MFs were separated by preparative SDS-PAGE (12.75% or 10% for GRF and ARA6), plotted onto PVDF membranes cut into 3 mm strips and detected with the respective antibodies. 10 μg protein per strip. Molecular weight markers are given in kDa. Arrow heads indicate the expected position of the respective protein.

Additionally, the plasma membrane H^+^ ATPase was also detected in the *Chara* membrane fraction by immunodetection with a major band slightly above 100 kDa (Fig 2A; for entire membranes of Western blots see S8 Fig). The observed double band for the PM H^+^ ATPase in the immunoblots (see below) might be caused by the different isoforms predicted by sequence alignment (S7 Fig), or the C-terminus that is known to be sensitive to degradation during sample preparation for SDS-PAGE, might have been cleaved off despite the precautions made during this step (see Materials and Methods). Other proteins that were putative markers for cell organelles or the cytosol were also tested by immunodetection: VHA-ɛ and H^+^ PPase as tonoplast markers, BiP2 for ER, ARA6 as a marker for plasma membrane and endocytic organelles [38], GRFs (14-3-3 proteins) and tubulin as cytosol markers (Fig 2B). The immunodetection experiment showed the suitability of the tested antibodies for *Chara* and confirmed the protein composition of the *Chara* membrane preparation containing membrane as well as membrane-associated proteins.

**Fig 3:**
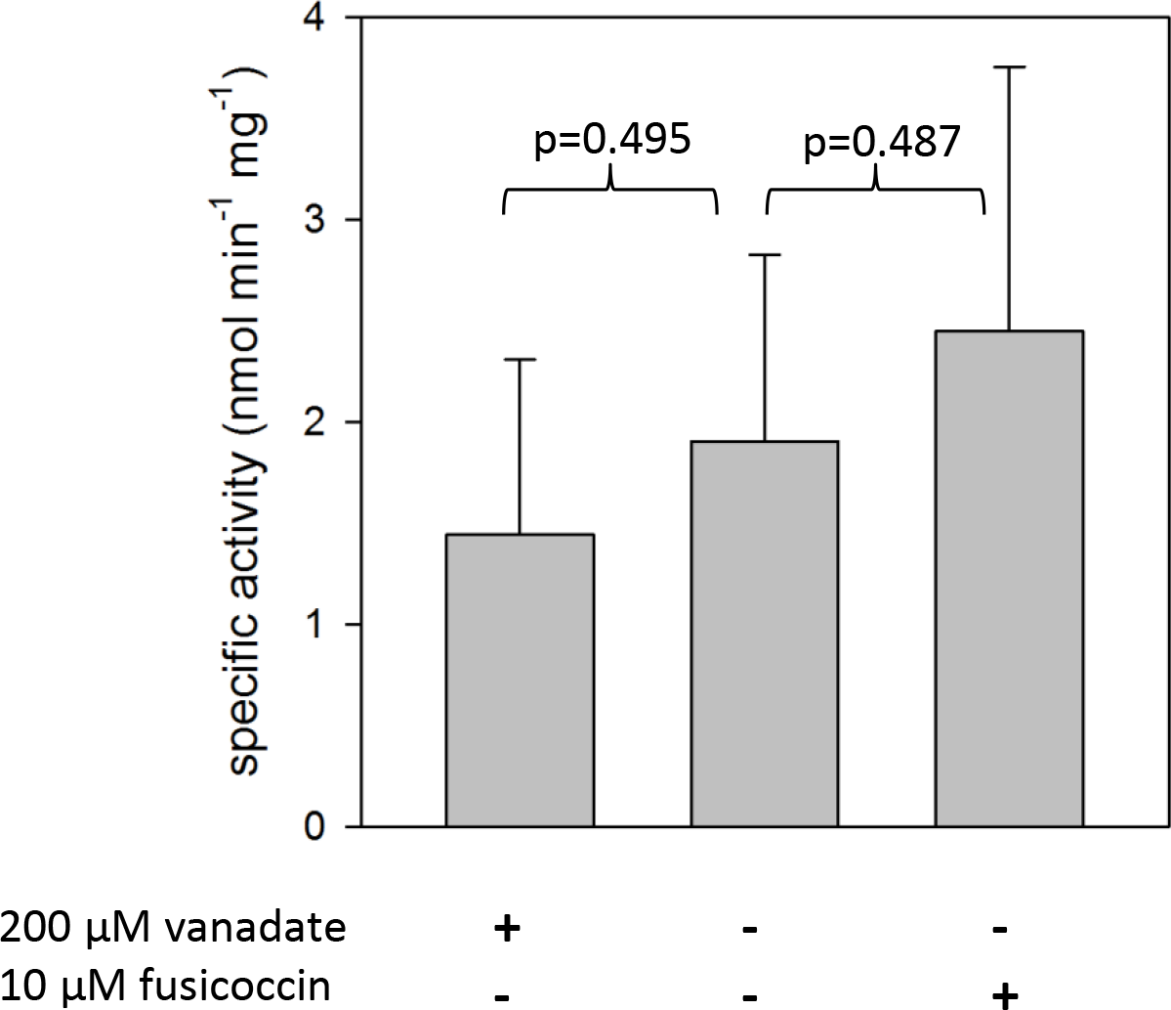
ATP hydrolysis activity in *Chara* MF. The specific ATP (3 mM Mg-ATP) hydrolysis activity was determined by detecting the released phosphate in the presence of 1 mM azide and 100 nM bafilomycin A to inhibit F- and V-type ATPases. Vanadate and fusicoccin were added to inhibit and to stimulate P-type ATPases respectively. Mean ± S.D. of 3–6 experiments. Probabilities (p) were determined with Student’s t-test.

Although GRFs/14-3-3 proteins were associated with *Chara* membranes (Fig 2B), the C-terminus of the *Chara* PM H^+^ ATPases coding for regulatory domains in higher plants was very different from higher plant sequences without any 14-3-3 binding motif (S7 Fig). The fungal toxin fusicoccin, which usually stimulates higher plant PM H^+^ ATPase by irreversibly binding 14-3-3 protein to the regulatory C-terminus, slightly, but not significantly, increased the ATP hydrolysis activity of *Chara* MF vesicles (Fig 3; compare [45]). A specific activity of 1.90 ± 0.92 nmol P_i_ min^-1^ mg^-1^ protein was measured, which could be inhibited by ca. 25% with 250 μM vanadate (1.44 ± 0.86 nmol P_i_ min^-1^ mg^-1^ protein) and stimulated by 25% using 10 μM fusicoccin (2.45 ± 1.31 nmol P_i_ min^-1^ mg^-1^ protein). The surprisingly low inhibition by vanadate has been reported previously in several *Chara* species [12, 46–48]. This difference in activity between the *Chara* PM H^+^ ATPase and those of higher plants may result from the observed sequence differences. The *Chara* PM H^+^ ATPases clustered together with other Chlorophyta P-type H^+^ ATPases from *Chlamydomonas reinhardtii*, *Volvox carteri f*. *nagariensis* and *Chlorella variabilis* but not with Bryophyta like *Physcomitrella patens* or *Marchantia polymorpha* (S9 Fig). This is in agreement with the results of Falhof et al [49] which indicate that 14-3-3-mediated regulation of PM H^+^ ATPases might have evolved with the occurrence of the first land plants.

### Membrane proteome in acidic and alkaline regions

**Fig 4:**
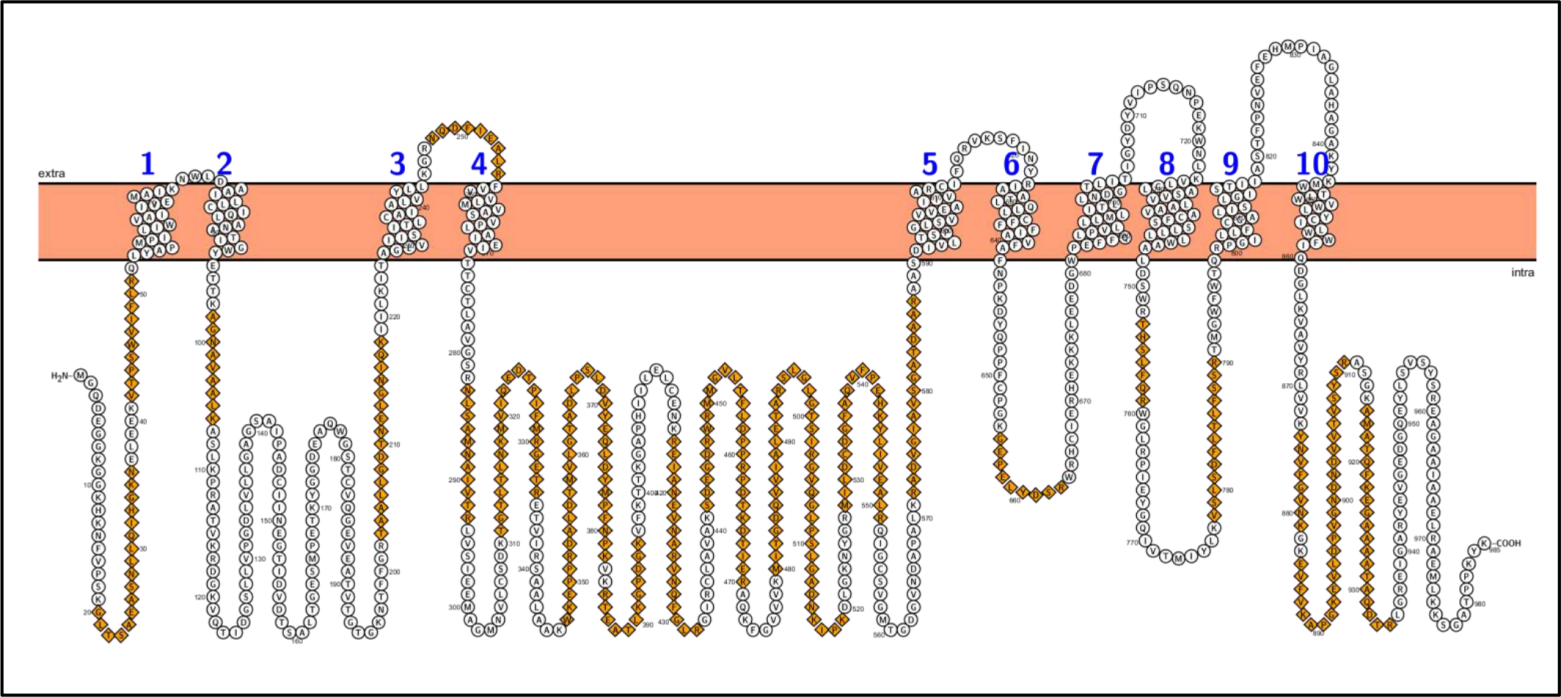
Topology of the *Chara* PM H^+^ ATPase. The sequence of unigene CL2034.contig1 was translated into amino acid sequence. Transmembrane domains were predicted by TMPred ([50]; (www.EXPASY.org)) and drawn with PROTTER (wlab.ethz.ch/protter). Peptides identified by mass spectrometry analysis, are given as orange diamonds.

**Fig 5:**
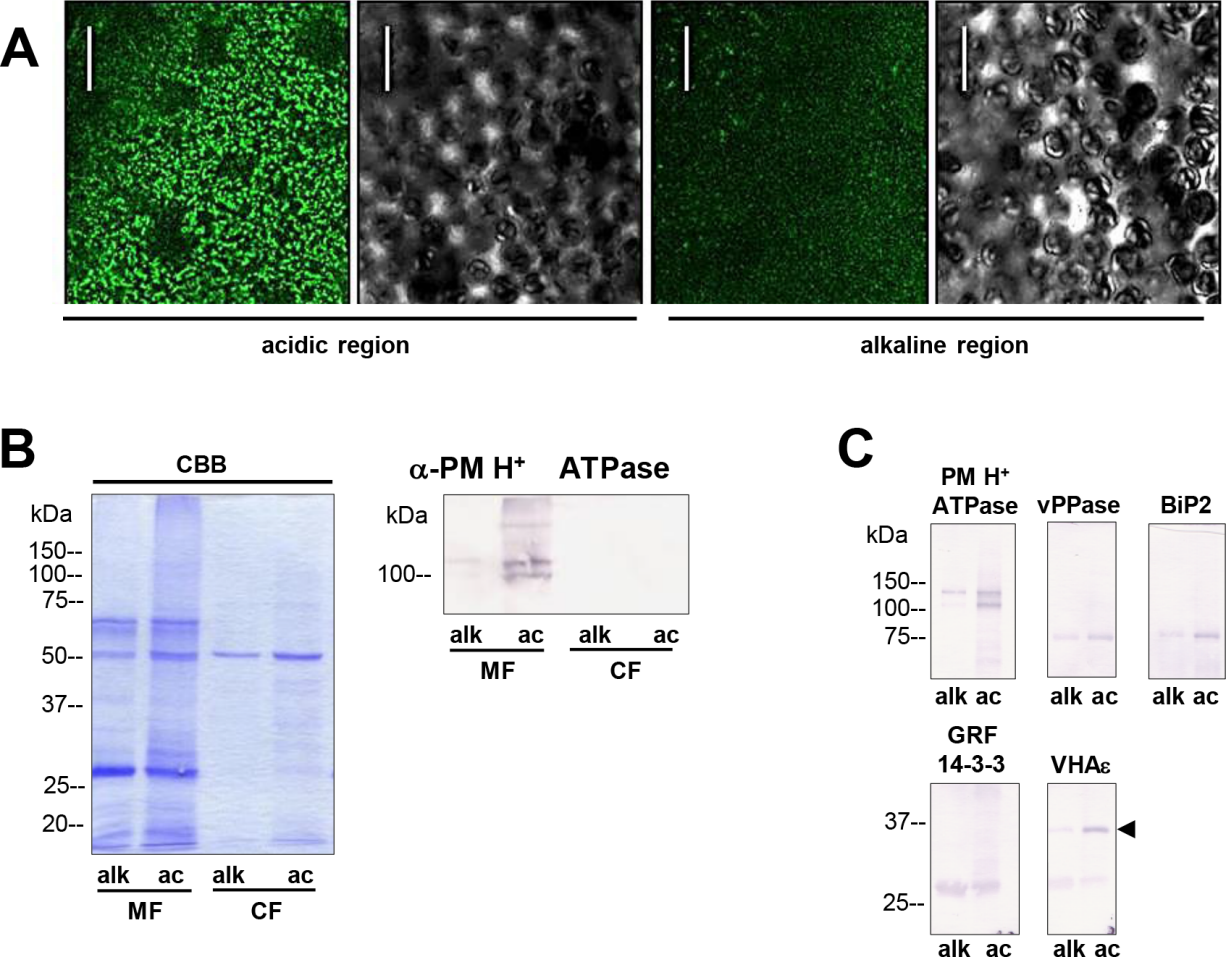
Differences in charasome abundance and protein expression in alkaline and acidic regions of *Chara* cells. (A) Left image pair: FM1-43-labelled charasomes (green fluorescence) and chloroplasts (bright field image) at an acidic band. Right image pair: FM1-43-stained charasomes are absent from the alkaline band; the bright field images show the chloroplasts. An FM1-43-stained internodal cell was cut into acid and alkaline regions as described in Materials and Methods guided by pH banding pattern visualized by phenol red. Cell fragments were mounted in artificial fresh water and examined in the CLSM. Bar = 20 μm (B) Proteins of membrane fractions (MF) and cytosolic fractions (CF) obtained from acidic (ac) and alkaline (alk) regions were separated by SDS-PAGE (10%) and stained with Comassie Brilliant Blue (CBB) or blotted onto PVDF membranes for immunodetection of the plasma membrane H^+^ ATPase using only the upper part of the membrane. 15 μg protein was loaded per lane. Numbers on the left refer to molecular weight markers in kDa. (C) Immunodetection of selected higher and lower molecular weight proteins from the membrane fraction (MF) as indicated. 10 μg protein was loaded per lane, 10% gel. PVDF membrane was cut into upper and lower halves at 50 kDa. Molecular weight markers given in kDa.

To identify and to annotate the peptides resulting from mass spectrometry analysis, the transcriptome data were translated into amino acid sequences and this custom-made *Chara* database was browsed for similar peptides identified by MS. Identified peptides were finally annotated to a physiological function on basis of their corresponding unigenes. The results are presented in the supplements section (S3 and S4 Tables) listing the peptide sequence with physical parameters (charge, m/z, mass, intensity) as well as orthologous genes from *Arabidopsis* and the corresponding BIN class. The coverage of the PM H^+^ ATPase (CL2034.contig1) with MS-derived peptides was examined (yellow marked peptides in S3 Table). Identified peptides were marked in a topology diagram of the *Chara* H^+^ ATPase (Fig 4) resulting in 35% coverage. A similar quality check was performed for the membrane proteomes prepared from the acidic and alkaline regions, respectively, and organelle proteins were detected via immunodetection using the antibody combinations tested and optimised for the entire *Chara* MF. It should be noted that the distribution of charasomes between acidic (enriched) and alkaline (depleted) regions was conserved during the separation step (Fig 5A). Staining of the whole membrane protein preparations showed only little difference between the alkaline and acidic MF with some additional protein bands in the acidic MF (Fig 5B, CBB). However, the PM H^+^ ATPase was much more abundant in the acidic than in the alkaline MF as expected (Fig 5B). No membrane contamination or PM H^+^ ATPase bands were observed in the soluble protein fraction (cytosolic fraction, CF). Likewise, the VHA-ɛ was more abundant in the acidic MF, whereas the vacuolar H^+^ PPase and the ER protein BiP2 showed only little differences and GRFs were equally distributed between acidic and alkaline MFs (Fig 5C).

**Fig 6:**
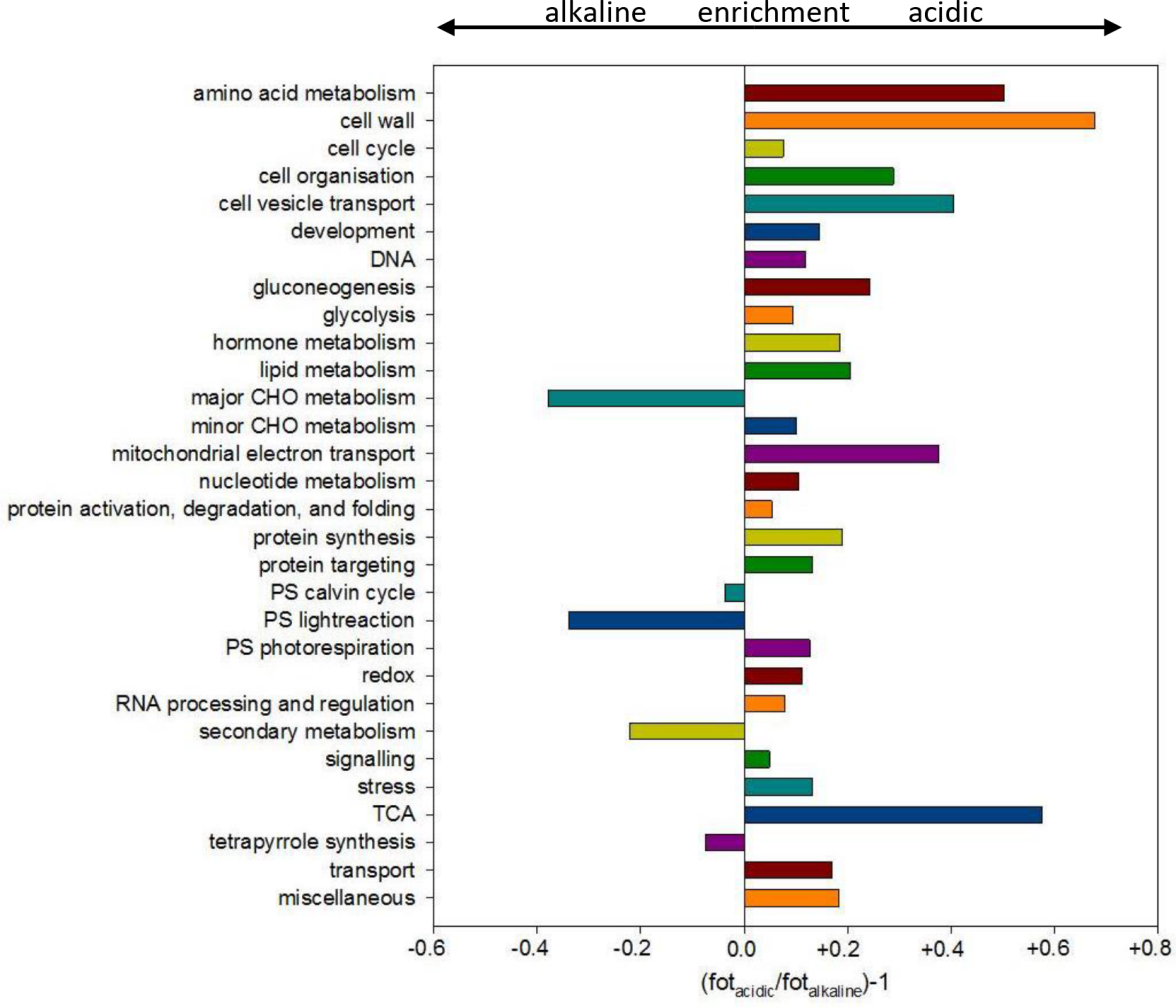
Differential proteome of the alkaline and acidic regions of *Chara* cells. Peptides were searched against the custom-made unigene *Chara* database (see also S1 Table) and assorted into the appropriated BIN classes. The fraction of total peptides (fot) for the respective BIN class was calculated. Ratios were subtracted by 1 to indicate enriched classes and depleted classes in the acidic regions by positive and negative values, respectively. CHO = carbohydrate metabolism, TCA = tricarboxylic acid cycle, PS = photosynthesis.

In addition to this restricted observation of only a few proteins, the proteome analysis enables a comprehensive view at the distribution of all identifiable peptides (proteins). All peptides from the acidic and alkaline membrane preparations using the *Chara* transcriptome database are listed in S4 Table with the corresponding *Chara* unigenes and functional BIN class. In contrast to *Arabidopsis* proteome studies where the identified peptides can be annotated faultlessly and exactly to a specific gene with a subsequent quantitative analysis of expression levels and abundances with sophisticated statistical validation, the non-sequenced *Chara* allows only a more coarse, semi-quantitative analysis by comparing the occurrence/frequency of peptides in specific functional classes, expressed as the fraction of total number of peptides (fot). Nevertheless, a clear tendency became visible when fot ratios were calculated for specific functional BIN classes (Fig 6). The proteins belonging to the functional categories ‘*amino acid metabolism*’, ‘*cell wall*’, ‘*cell vesicle transport*’, ‘*mitochondrial electron transport*’ and ‘*tricarbonic acid cycle (TCA)*’ were more abundant in the acidic than in the alkaline regions. In alkaline regions higher amounts of peptides from the categories ‘*major carbohydrate metabolism (CHO)’*, ‘*photosynthesis (PS) lightreaction*’ and ‘*secondary metabolism*’ could be noticed, thus indicating a probable separation of physiological functions between the acidic and alkaline regions in the single, individual *Chara* cell. Individual proteins from the functional classes ‘*34 transport*” and ‘*31*.*4 cell*.*vesicle transport*’ are further listed in Tables 1 and 2, respectively. Most proteins exhibit a higher spectral count in the acidic than in the alkaline MF, e.g. 40% more PM H^+^ ATPases, 45% more clathrin heavy chain, 62% more adaptins and up to 236% more coatamers (coatamer beta subunit).

**Table 1:** List of identified proteins of the BIN class ‘*34 transport*’ by LC-MS/MS.

		No. of spectral counts				
BIN class	Protein description (TAIR10)	Total	MF acidic	MF alkaline	Accession no. (TAIR 10)	Sub-cellular localization (SUBA4)
34.1 transport.p- and v-ATPases		**711**	**415**	**296**		
	aminophospholipid ATPase 3 (ALA3)	7	6	1	at1g59820	Golgi, PM
	plasma membrane H^+^ ATPase 3(HA3)	694	404	290	at5g57350	PM
	plasma membrane H^+^ ATPase 4 (HA4)	10	5	5	at3g47950	PM
34.1.1 transport.p- & v-ATPases.H^+^-transporting two-sector ATPase		**1469**	**821**	**648**		
	V-ATPase subunit A (VHA-A)	394	223	171	at1g78900	V, Golgi
	V-ATPase subunit A(VHA-A2)	109	66	43	at2g21410	V
	V-ATPase subunit C (VHA-C)	117	61	56	at1g12840	V, Golgi
	ATPase, V0/A0 complex, subunit C/D	105	61	44	at3g28710	V
	vacuolar ATPase subunit F family protein	35	21	14	at4g02620	V
	vacuolar ATP synthase subunit E1	130	65	65	at4g11150	V, Golgi
	vacuolar H^+^-pumping ATPase 16 kDa proteolipid (AVA-P1)	74	40	34	at4g34720	V
	ATPase, V1 complex, subunit B protein	348	193	155	at4g38510	V
	ATPase, V1 complex, subunit H protein	157	91	66	at3g42050	V, Golgi, PM
34.2 transport.porins		**346**	**224**	**122**		
	translocase of the outer mitochondrial membrane 40 (TOM40)	27	22	5	at3g20000	M
	voltage dependent anion channel 1 (VDAC1)	319	202	117	at3g01280	M
34.2 transport.sugars		**157**	**77**	**80**		
	major facilitator superfamily protein	10	5	5	at1g19450	V
	major facilitator superfamily protein	39	19	20	at5g59250	P
	major facilitator superfamily protein	1	1	0	at5g64500	V
	oligosaccharyltransferase/magnesium transporter protein	10	6	4	at1g61790	ER
	plastidic glucose translocator (PGLCT)	85	38	47	at5g16150	P
	sucrose transporter 2 (SUT2)	12	8	4	at2g02860	Golgi, V, PM
34.3 transport.H^+^ transporting pyrophosphatase		**573**	**315**	**258**		
	vacuolar H^+^- pyrophosphatase 1 (AVP1)	570	313	257	at1g15690	V
	vacuolar H^+^- pyrophosphatase 2 (AVP2)	3	2	1	at1g78920	G
34.5 transport.ammonium		**177**	**95**	**82**		
	ammonium transporter 1;2 (AMT1;2)	58	23	35	at1g64780	PM
	ammonium transporter 1;5 (AMT1;5)	105	60	45	at3g24290	PM, V
	P-loop containing nucleoside triphosphate hydrolases protein	14	12	2	at1g72660	C
34.6 transport.sulphate		**9**	**8**	**1**		
	sulfate transporter 1;2 (SULTR1;2)	9	8	1	at1g78000	PM
34.8 transport.metabolite transporters at the envelope membrane		**67**	**37**	**30**		
	EamA-like transporter family protein	28	19	9	at1g77610	V, PM
	glucose-6-phosphate/phosphate translocator 2 (GPT2)	18	9	9	at1g61800	P
	glucose-6-phosphate/phosphate translocator-related	21	9	12	at5g33320	P
34.9 transport.metabolite transporters at the mito membrane		**434**	**250**	**184**		
	adenine nucleotide transporter 1 (ADNT1)	9	9	0	at4g01100	M
	dicarboxylate transport 2.1 (DIT2.1)	10	5	5	at5g64290	P
	mitochondrial substrate carrier family protein	11	6	5	at1g07030	M
	mitochondrial substrate carrier family protein	161	88	73	at5g19760	M
	mitochondrial substrate carrier family protein	8	8	0	at5g46800	M
	mitochondrial substrate carrier family protein	12	8	4	at5g48970	M
	phosphate transporter 3;1 (PHT3;1)	158	95	63	at5g14040	M
	TLC ATP/ADP transporter (ATNTT2)	65	31	34	at1g15500	P
34.12 transport.metal		**29**	**19**	**10**		
	cation/H^+^ exchanger 19 (CHX19)	17	9	8	at3g17630	PM
	cation/H^+^ exchanger 18 (CHX18)	9	7	2	at5g41610	PM
	heavy metal atpase 2 (HMA2)	1	1	0	at4g30110	PM
	sodium:hydrogen antiporter 1 (NHD1)	2	2	0	at3g19490	P
34.14 transport.unspecified cations		**399**	**263**	**136**		
	ADP/ATP carrier 2 (AAC2)	10	5	5	at5g13490	M
	equilibrative nucleotide transporter 1 (ENT1)	7	6	1	at1g70330	PM
	high-affinity K^+^ transporter 1 (HKT1)	2	2	0	at4g10310	PM
	Na^+^/H^+^ exchanger 1 (NHX1)	8	7	1	at5g27150	V, PM
	mitochondrial substrate carrier family protein	295	202	93	at5g56450	PM
	solute:sodium symporter (DUR3)	77	41	36	at5g45380	PM
34.15 transport.potassium		**53**	**27**	**26**		
	Ca^2+^ activated outward rectifying K^+^ channel 5 (KCO5)	23	13	10	at4g01840	V
	K^+^ efflux antiporter 2 (KEA2)	30	14	16	at4g00630	P
34.16 transport.ABC transporters & multidrug resistance systems		**425**	**253**	**172**		
	ABC2 homolog 6 (ATH6)	27	20	7	at3g47780	PM
	ABC2 homolog 7 (ATH7)	29	17	12	at3g47790	PM
	ABC transporter family protein	3	3	0	at5g60740	PM
	ABC transporter of the mitochondrion 1 (ATM1)	1	1	0	at4g28630	M
	ABC transporter of the mitochondrion 3 (ATM3)	6	5	1	at5g58270	M
	ABC-2 type transporter family protein	36	21	15	at2g01320	P
	ATP-binding cassette A1 (ABCA1)	7	7	0	at2g41700	V, M, Golgi
	ATP-binding cassette A2 (ABCA2)	85	56	29	at3g47730	PM
	ATP binding cassette B1 (ABCB1)	41	25	16	at2g36910	PM
	general control non-repressible 4 (GCN4)	39	22	17	at3g54540	N
	general control non-repressible 5 (GCN5)	6	4	2	at5g64840	PM
	multidrug resistance-associated protein 1 (MRP1)	20	19	1	at1g30400	V
	multidrug resistance-associated protein 2 (MRP2)	12	6	6	at2g34660	V
	multidrug resistance-associated protein 12 (MRP12)	2	2	0	at1g30420	V, PM
	multidrug resistance-associated protein 13 (MRP13)	3	3	0	at1g30410	V
	non-intrinsic ABC protein 3 (NAP3)	1	1	0	at1g67940	V
	non-intrinsic ABC protein 6 (NAP6)	27	12	15	at1g32500	P
	non-intrinsic ABC protein 8 (NAP8)	56	20	36	at4g25450	P
	P-glycoprotein 9 (PGP9)	8	6	2	at4g18050	PM, V
	P-glycoprotein 11 (PGP11)	2	1	1	at1g02520	PM
	protein kinase superfamily protein	9	1	8	at1g79600	P
	protein kinase superfamily protein	5	1	4	at4g31390	P
34.18 transport.unspecified anions		**124**	**85**	**39**		
	cation-chloride co-transporter 1 (CCC1)	5	3	2	at1g30450	Golgi
	chloride channel E (CLC-E)	4		4	at4g35440	P
	chloride channel D (CLC-D)	57	45	12	at5g26240	Golgi
	P-loop nucleoside triphosphate hydrolases superfamily protein	8	7	1	at1g01910	Golgi
	P-loop nucleoside triphosphate hydrolases superfamily protein	50	30	20	at3g10350	P
34.19 transport.Major Intrinsic Proteins		**222**	**116**	**106**		
	beta-tonoplast intrinsic protein (BETA-TIP)	162	83	79	at1g17810	V
	tonoplast intrinsic protein 2.1 (OsTIP2.1)	37	19	18	q7xa61|tip21_orysa	V
	small and basic intrinsic protein 1A (SIP1A)	23	14	9	at3g04090	ER
34.21 transport.calcium		**195**	**93**	**102**		
	CAX-interacting protein 2 (CXIP2)	20	8	12	at2g38270	P
	endomembrane-type CA-ATPase 4 (ECA4)	3	3	0	at1g07670	ER, PM
	ER-type Ca^2+^-ATPase 1 (ECA1)	75	31	44	at1g07810	ER, PM, V
34.99 transport. Misc		**163**	**96**	**67**		
	emp24/gp25L/p24 family/GOLD family protein	78	45	33	at1g09580	PM
	plasma-membrane choline transporter family protein	20	13	7	at1g25500	P, PM
	SecY protein transport family protein	44	24	20	at1g29310	ER
	secretory carrier 3 (SC3)	11	9	2	at1g61250	PM
	major facilitator superfamily protein	10	5	5	at3g13050	PM, V
		**5553**	**3194**	**2359**		

Fragmentation spectra from *Chara australis* microsomes (MF_acidic_ and MF_alkaline_) were searched against an in house-made database containing *Chara australis* protein amino acid sequences generated from the *Chara australis* transcriptome. *Arabidopsis thaliana* orthologous genes are listed. C = cytosol, ER = endoplasmic reticulum, Golgi = Golgi apparatus, M = mitochondrium, N = nucleus, P = plastid, PM = plasma membrane, V = vacuole

**Table 2:** List of identified proteins belonging to the BIN class ‘*31*.*4 cell*.*vesicle transport’*.

		No. of spectral counts				
BIN class	Protein description (TAIR10)	Total	MF acidic	MF alkaline	Accession no.	Sub-cellular localization (SUBA4)
31.4 cell.vesicle transport						
	adaptin family protein	112	69	43	at4g11380, at5g11490	PM, Golgi
	adaptor protein complex AP-4, epsilon subunit	3	3	0	at1g31730	N, C
	alpha-adaptin (alpha-ADR)	89	55	34	at5g22770	PM
	BEACH-domain-type protein	9	6	3	at1g03060	C, N
	chaperone DnaJ-domain superfamily protein	29	15	14	at4g12780	N
	clathrin adaptor complex small chain family protein	2	2	0	at4g35410	M
	clathrin adaptor complex medium subunit family protein	46	26	20	at5g05010	Golgi
	clathrin, heavy chain	473	280	193	at3g08530	PM, C
	coatomer, alpha subunit	155	96	59	at1g62020	C, Golgi
	coatomer, beta subunit	165	116	49	at1g52360, at4g31480, at3g15980	C, Golgi
	coatomer, epsilon subunit	20	14	6	at1g30630	Golgi
	coatomer, gamma-2 subunit	75	53	22	at4g34450	C
	emp24/gp25L/p24 family/GOLD family protein	22	12	10	at3g22845	ER, Golgi
	exocyst complex component sec15A (SEC15A)	6	6	0	at3g56640	PM, C
	exocyst subunit exo70 family protein A2 (EXO70A2)	3		3	at5g52340	C, PM
	novel plant snare 11 (NPSN11)	44	27	17	at2g35190	PM
	Rer1 family protein	10	6	4	at4g39220	ER, Golgi
	Sec23/Sec24 protein transport family protein	3	3	0	at4g32640	C, N
	SH3 domain-containing protein	20	19	1	at4g34660	C
	SNARE-like superfamily protein	18	10	8	at1g47830, at4g08520	C
	sol. N-ethylmaleimide-sens. adaptor protein 30 (SNAP30)	6	5	1	at1g13890	N, C
	sorting nexin 2A (SNX2a)	1	1	0	at5g58440	Golgi
	structural molecules	2	2	0	at1g79990	ER
	syntaxin of plants 131 (SYP131)	47	27	20	at3g03800	PM
	syntaxin of plants 132 (SYP132)	17	11	6	at5g08080	PM
	syntaxin of plants 71 (SYP71)	46	26	20	at3g09740	PM
	syntaxin/t-SNARE family protein	2	1	1	at5g46860	V
	target of Myb protein 1	11	9	2	at5g16880	C
	vesicle transport v-SNARE family protein	18	10	8	at5g39510	V
	vesicle-associated membrane protein 714 (VAMP714)	83	45	38	at5g22360	Golgi
	vesicle-associated membrane protein 721 (VAMP721)	79	45	34	at1g04750	PM
	vesicle-associated membrane protein 725 (VAMP725)	2	2	0	at2g32670	PM
		**1618**	**1002**	**616**		

Fragmentation spectra from *Chara australis* microsomes (MF_acidic_ and MF_alkaline_) were searched against an in house-made database containing *Chara australis* protein amino acid sequences generated from the *Chara australis* transcriptome. *Arabidopsis thaliana* orthologous genes are listed. C = cytosol, ER = endoplasmic reticulum, Golgi = Golgi apparatus, M = mitochondrium, N = nucleus, P = plastid, PM = plasma membrane, V = vacuole

**Fig 7:**
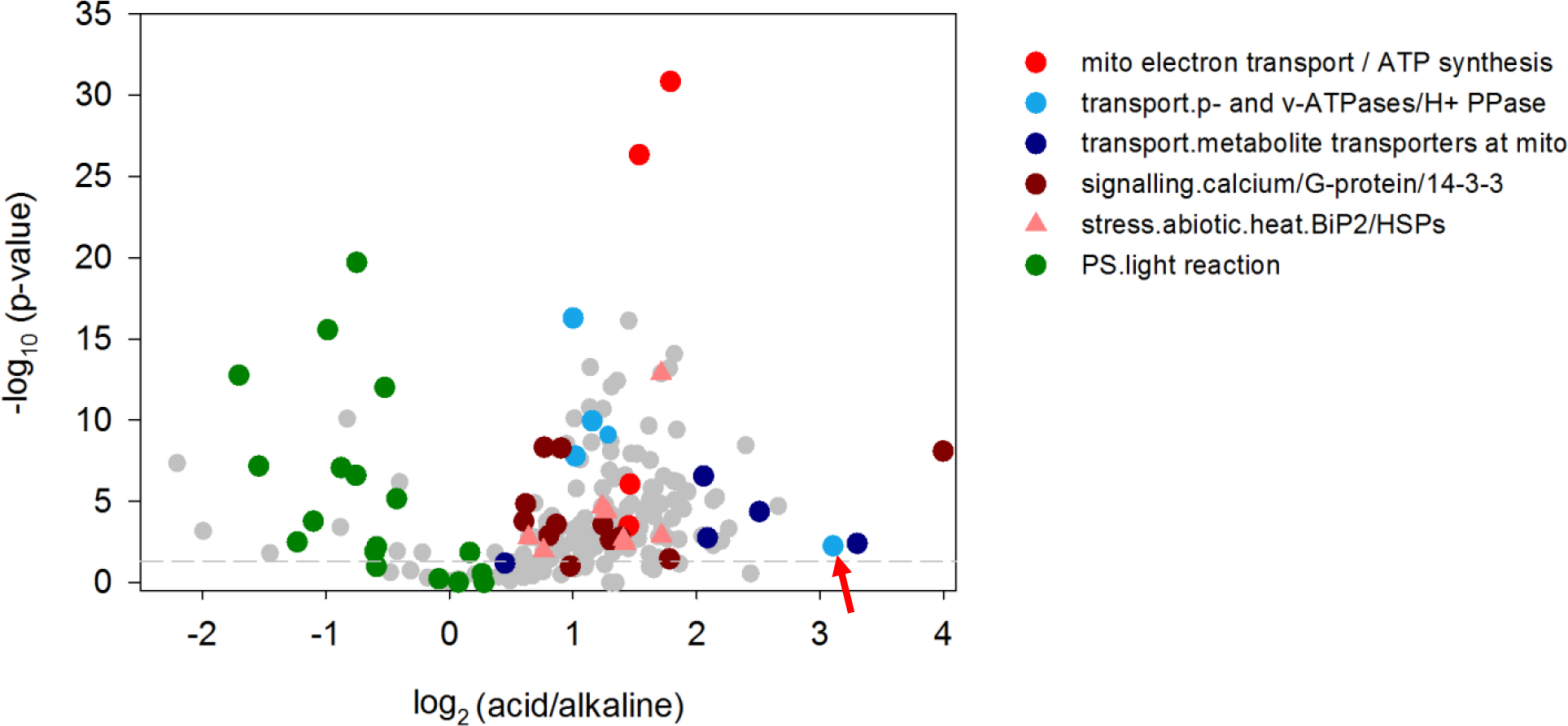
Distribution of individual peptides of the alkaline and acidic regions of *Chara* cells. Peptides were annotated using TAIR database and categorized into BIN classes as indicated. Differential expression was analysed with cRACKER software. Dotted line shows threshold of p = 0.05. Arrow indicates the PM H^+^ ATPase peptide.

Furthermore, a detailed analysis of specific peptides present in the acidic as well as in the alkaline MF preparations was performed by a search against the TAIR database resulting in the intensity values of these peptides and the annotation to their corresponding, orthologous *Arabidopsis* genes, followed by a statistical analysis using cRACKER [43]. The results are shown in Fig 7 with each dot presenting a peptide and coloured dots belonging to the BIN class as indicated. Again, peptides belonging to the category ‘PS.lightreaction’ showed lower expression in the acidic than in the alkaline regions (negative log2 ratios) whereas peptides belonging to the categories ‘*transport*’, ‘*mitochondrial electron transport*’ but also ‘*signalling*.*calcium*’ and ‘*stress*.*abiotic*.*heat*.*BiP2*’ showed higher abundances in the acidic regions thus confirming the previous results from immunodetection and searches in the custom-made *Chara* database.

Since charasomes and mitochondria are highly abundant in the acidic regions one might assume that the identified membrane proteins were localised in the charasome membrane and in the mitochondrial membranes, respectively. Surprisingly, not only plasma membrane and mitochondrial transporter proteins but also tonoplast membrane proteins showed higher abundances in the acidic regions. In addition, a large number of proteins involved in vesicle trafficking and fusion indicating a higher activity of membrane transport in the charasome-rich regions as compared with charasome-poor areas.

## Discussion

In this study the different composition of membrane proteins in acidic and alkaline regions, which were localised side by side in the same internodal cells of *Chara australis* R. Br., was investigated by shotgun proteomics. The here presented techniques and the obtained public-available, functionally-annotated sequence database may allow further molecular biology research on a valuable model organism that was hitherto investigated mainly with “classical” physiological methods [51]. The proteome study, supported by immunodetection, identified several membrane proteins associated with acidic regions that contain a high density of specific plasma membrane invaginations, the charasomes. Some of these data confirm earlier studies, suggesting that the methods applied here are suited to detect spatial differences in the protein composition of single cells.

### Methodological approach

Identification of the molecular composition of charasomes contains at least two major challenges to be mastered: i) the extremely low amount of starting material (see Fig 1B for the small alkaline bands) and preservation of the charasome distribution during MF preparation and ii) *Chara australis* is a non-genome-sequenced organism with scarce sequence information with almost no annotated functions. The first problem could be solved by careful collection of internodal cell regions and precise preparation of membrane proteins by down-scaling (low volume) the MF preparation and in-solution digestion as well as highly sensitive mass spectrometry analysis using only 5 μg protein per analysis run [52]. The second challenge was to provide more sequence information with reliable and robust annotation of *Chara* transcripts leading to a new RNAseq analysis and annotation of the unigenes with Mercator software in addition to the standard bioinformatics provided by the company. Both sequence data sets were transformed into a single database suitable for peptide identification and annotation using MaxQuant software for the proteome analysis. However, the challenge of translating the omics results into physiology still remains [53]. Although the presented data may also provide a solid base for other open questions on *Chara* physiology, development or evolution, the following analysis and discussion will be focussed on the physiological role of charasomes and acidic/alkaline banding pattern.

For this, the identified unigenes were classified into functional categories (MapMan BINs) but not annotated to their corresponding genes as can be done for full-genome-sequenced organisms. Two strategies for analysing the difference of the classified proteome data were used: firstly, the fraction of total peptides (fot) was calculated from the custom-made *Chara* database (Fig 6) whereas peptide intensity ratios were calculated from results of the search against TAIR (Fig 7), which also allowed further statistical analysis of the distribution of single peptides between acidic and alkaline regions. In general, both data analyses provide similar results, e.g. the category ‘transport’ is more abundant in acidic regions (Fig 6, Table 1) just like the peptides of transport proteins (cyan dots in Fig 7) whereas photosynthetic peptides/proteins are more abundant in alkaline regions.

The differences in membrane proteins between acidic and alkaline regions were not due to the increased plasma membrane area provided by charasomes. All proteome data refer to equal protein amounts in the membrane fractions prepared from acidic and alkaline regions, and semi-quantitation (spectral counts, fraction of total) was therefore independent of the surface areas of the respective regions.

### PM H^+^ ATPase, mitochondrial and other proteins enriched in acidic regions

The distribution of specific proteins mainly confirmed earlier microscopic observations at the molecular level. Particularly for the PM H^+^ ATPase, a high activity was reported to be associated with mature charasomes [19] and immunolabelling of cells with an antibody against the PM H^+^ ATPase has shown a high abundance of this transporter in charasome membranes [3]. Consistent with these findings, the PM H^+^ ATPase was much more abundant in membrane fractions of acidic regions. On the other hand, 14-3-3 proteins, which are major regulators of PM H^+^ ATPase activity in cells of higher plants [54–56], were not enriched at acidic regions, and their role in the activation of *Chara* PM H^+^ ATPases remains enigmatic. Comparison with the well-known *Arabidopsis* AHA2 and other higher plant PM H^+^ ATPase sequences revealed an unusual C-terminus for *Chara* ATPases, e.g. the prominent 14-3-3 binding motif (phospho(S/T)X_1-2_-COOH is missing. In higher plants the C-terminus functions as a regulatory domain to modulate the H^+^ transport activity upon phosphorylation of threonine and serine residues, and thus affects the binding of 14-3-3 proteins [56, 57]. The absence of the 14-3-3 binding motif in the *Chara* plasma membrane H^+^ ATPase is consistent with the lack of a significant stimulation of vanadate-sensitive ATP hydrolysis upon addition of fusicocccin [45]. The activation of the outward directed H^+^ transport observed within one to several hours after exposure of internodal cells to fusicoccin may reflect an indirect effect of 14-3-3 proteins via other ion transporters on the H^+^ ATPase of *Chara* [48].

**Fig 8:**
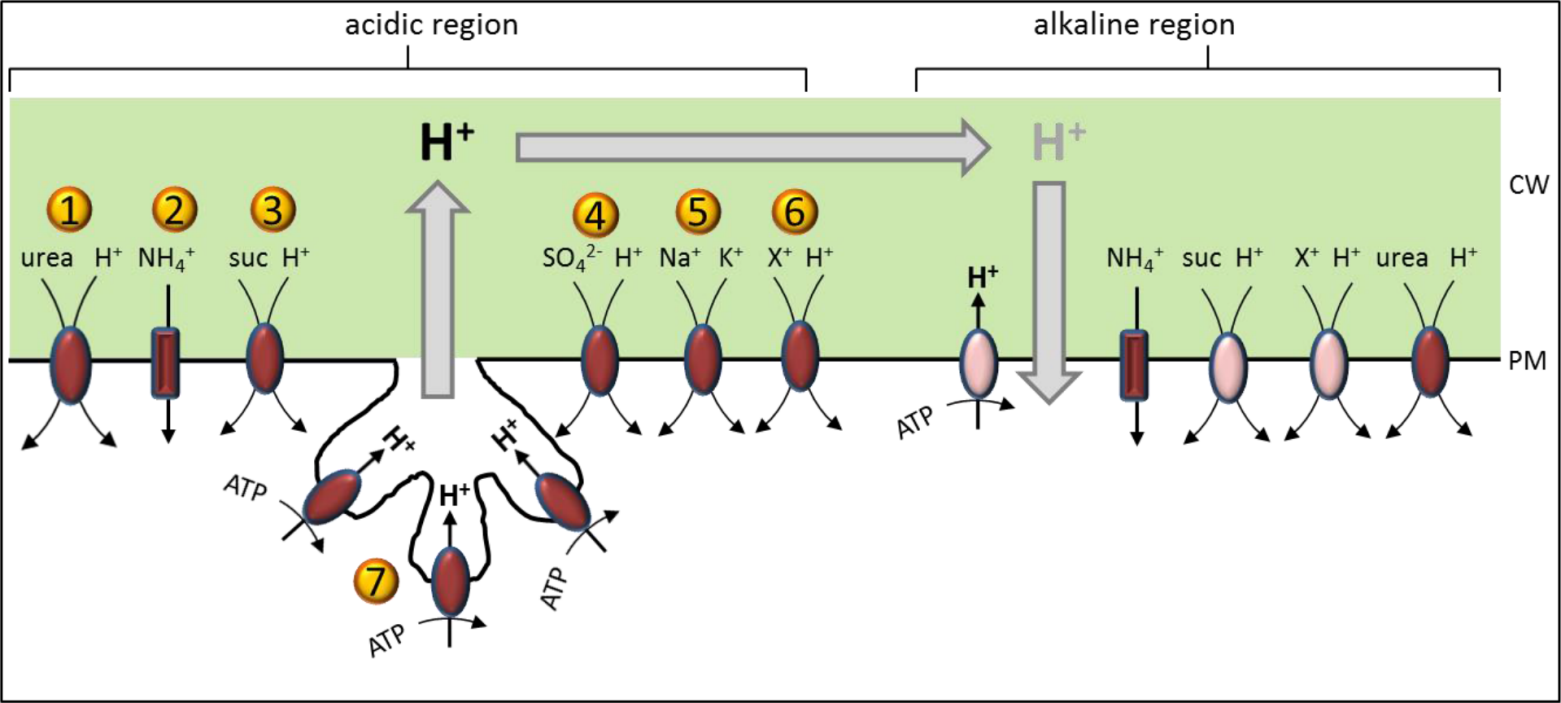
Distribution of H^+^ transporters between acidic and alkaline regions. H^+^ transporting proteins predicted for plasma membrane localisation (Table 1) were drawn in the scheme according to their abundance as dark red or bright red symbols representing high and low abundance, respectively. The cell wall (CW) is sectioned in acidic (H^+^ in black) or alkaline (H^+^ in grey) regions with the plasma membrane (PM) underneath and charasomes drawn as invaginations in the acidic region. The ion transporters are labelled as following: 1 = APC (amino acid/polyamine/organo-cation) transporter, urea/H^+^ symporter DUR3 (at5g45380) 2 = ammonium transporter (NH_4_^+^ or NH_3_ plus H^+^) AMT1;2 and AMT1;5 (at1g64780, at3g24290)3 = sucrose/H^+^ symporter SUC3/SUT2 (at2g02860) 4 = sulphate/H^+^ symporter SULTR1 (at1g78000) 5 = Na^+^/cation transporter HKT1 (at4g10310) 6 = cation/H^+^ exchanger CHX18/19 (at5g41610, at3g17630) 7 = P-type H^+^ ATPase, PM H^+^ ATPase (at5g57350).

The participation of PM H^+^ ATPases in the pH banding of characean internodal cells is undisputable, but the question of how the cells maintain cytoplasmic pH homoeostasis is still under debate. Electrophysiological measurements suggest that H^+^ export at the acidic region is balanced by an efflux of OH^-^ at the alkaline bands but the necessary OH^-^/H^+^ channels have not been identified so far [2, 35]. A possible alternative is a large net H^+^ influx via symporter or antiporter preferentially localised in alkaline regions. Fig 8 shows the distribution of transporters according to the data of this study (Table 1). No proteins or transcripts for an OH^-^/H^+^ channel could be identified in the present study, too, and the H^+^ carriers are either equally abundant or even less frequent at the alkaline regions. However, due to the low abundance of the PM H^+^ ATPase at the alkaline sections a large net uptake of H^+^ causes the local alkalinisation. In addition, it should be kept in mind that this first study may not give a complete view of all involved ion transporters, and that transporter activities depend not only on abundance but can be regulated via membrane potential and substrate (ion) concentration. For instance, proton symporters in an alkaline band might be inactive due to a depolarized membrane potential which lowers the proton motive force [58].

Cell regions with abundant charasomes are also significantly enriched in cortical mitochondria [3, 18]. Accordingly, proteins of the categories mitochondrial electron transport including F-type ATPases, mitochondrial metabolite transport as well as proteins associated with the tricarboxylic acid cycle (TCA) were among the highest upregulated proteins at acidic regions. However, not all mitochondrial transporters show increased abundance in the acidic regions. While translocases of the outer mitochondrial membrane (*Arabidopsis* orthologue TOM40, AT3G20000) and the voltage-dependent anion channel (VDAC1, AT3G01280) are at least doubled in acidic regions, some members of the mitochondrial substrate carrier family are equally distributed between the acidic and alkaline regions (Table 1). The spatio-temporal variations in the abundance of cortical mitochondria along an internodal cell are, however, not due to the presence of charasomes per se. Similar pH-dependent patterns occur in the charasome-free internodal cells of the characean genus *Nitella* where they probably reflect differences in photosynthetic activity of the chloroplast [59]. Likewise, the inconsistency in the abundance of mitochondrial membrane proteins may indicate that the physiological state of mitochondria probably differs between acidic and alkaline bands. For instance, the mitochondria in acidic regions are more active in respiration and ATP synthesis whereas mitochondria in the alkaline region preferably accumulate metabolites.

Our study also revealed upregulation of V-ATPases at acidic regions including V-ATPase subunit A. The VHA-A isoform VHA-a1 (At2g28520), has been found to localize in the TGN/early endosome [60, 61] and its high abundance at charasomes would be consistent with the formation of charasomes from TGN-derived material [25]. The identified *Chara* sequences were related to the *Arabidopsis* VHA-a isoforms At1g78900 and At2g21410, and at the present molecular resolution of the study, which mainly consists of partial sequences annotated to functional categories, it will be rough speculation to distinguish between closely related isoforms to attribute them to specific localization and cellular functions.

However, further studies are required to explain the higher abundance of the tonoplast (endomembrane)-associated V-ATPases in the proteome and in immunoblots of MF of acidic regions. The charasomes are separated from the tonoplast by the stationary chloroplast files and the streaming endoplasm. Tonoplast and endoplasm are unlikely to be preserved during mechanical separation of alkaline and acidic regions. It thus remains to be investigated where the ‘vacuolar’ V-ATPase is located within the charasome-enriched regions. A possible explanation is that this enzyme is not only located in the tonoplast of the central vacuole but also in the membrane of smaller vacuoles [62] or vesicles and that these organelles are perhaps more abundant at acidic regions in agreement with the enrichment of vesicle transport proteins in these areas. Furthermore, fusion of vacuoles or multivesicular bodies with the plasma membrane might occur during charasome formation hinting at an unconventional secretion pathway [63], and finally localizing the V-ATPase in the plasma membrane. Unfortunately, the antibody against VHA-ɛ used for immunoblots in this study is not suited for immunolocalisation in *Chara*. The upregulation of BiP2 at acidic regions is less enigmatic because of the close vicinity between charasomes and the cortical endoplasmic reticulum. Work is now in progress to study the distribution of the cortical endoplasmic reticulum in acid and alkaline region and its possible role in charasome formation and function. Support for a higher abundance of endoplasmic reticulum cisternae at acidic, charasome-rich regions comes also from the upregulation of COPI proteins (coatamer subunits) which are involved in retrograde transport from the Golgi bodies to the endoplasmic reticulum [64].

### High abundance of clathrin at acidic regions reflecting charasome turnover

The significant enrichment of clathrin heavy chains and clathrin adaptor proteins at the acidic regions is consistent with the important role of these proteins in the formation as well as in the degradation of charasomes [22, 65]. The formation of charasomes is light-dependent and under natural and culture conditions charasomes will be formed or degraded slowly, via a period of at least several days, and according to differences in light exposure due to (local) shading from the upper parts of the thallus. It thus appears reasonable that proteins involved in charasome turnover are enriched at acidic regions. The fact that charasome tubules may grow into the adjacent cell wall [18] as well as known differences in cell wall composition between acidic and alkaline regions [66] are likely to correlate with the upregulation of cell wall proteins at charasome-rich regions. The upregulation of a Ca^2+^ binding protein in acidic regions is interesting because charasomes can be labeled by chlortetracycline (unpublished own data), which has been used as a fluorescent probe of membrane-associated calcium [67–69].

### Unexpected upregulation of photosynthesis-related proteins in alkaline regions

The present study also revealed the unexpected and rather surprising finding of the lower abundance of many photosynthesis proteins in the charasome-rich acidic region compared to the alkaline regions. One of the main function of charasomes is the acidification of the medium which leads to the conversion of membrane impermeable hydrogen carbonate, the dominant carbon species in neutral and alkaline water, into membrane permeable CO_2_. The enhanced availability of carbon is probably the main reason why the photosynthetic activity of chloroplasts, which are evenly distributed in the cell cortex, correlates with the pH banding pattern by being high at charasome-rich acidic and low at charasome-poor alkaline regions [14, 15]. In spite of that, chloroplasts from acidic regions of single cells contained far less proteins involved in photosynthetic light reactions or in the Calvin cycle. We carefully considered the possibility of a methodological bias. Under stress conditions, the pH banding pattern can be disturbed [70] and charasome-enriched regions might transiently become alkaline [35]. It is thus feasible that the pH banding pattern visualized by phenol red prior to dissection of cells does not always reflect the distribution of charasomes. However, we can exclude this possibility because in that case we would neither observe PM H^+^ ATPase nor mitochondrial proteins or clathrin to be upregulated at the acid regions. A possible explanation is that the upregulation of the photosynthesis-related proteins at alkaline regions represents a kind of compensation due to low availability of carbon dioxide. However, the different physiological states of chloroplasts and mitochondria in the acidic and alkaline regions need to be investigated in more detail.

In summary, the obtained protein sequence data of *Chara* presented in this study support the current view of the physiological compartmentation along internodal cells of *Chara* with the specific function of charasomes. The proteome data also suggest previously unrecognized, new roles of vacuolar and ER membranes/proteins in the formation or function of charasomes. Furthermore, the annotated transcript and protein sequences provide a valuable publicly available database for future research on *Chara australis* and other characean species.

## Supporting information

S1 Table Summary of sequence assembly data. Sequence lengths are given in nucleotide numbers (nt) and base pairs (bp) or in percent for GC content, unknown nucleotides (N) or high quality reads (Q20). Q20 indicates the percentage of reads with an error rate ≤ 1%. N50 = 350 means that 50% of the reads could be assembled into contigs (or unigenes) with lengths > 350 bp.
(PDF)

S2 Table Summary of functional annotation of unigenes. Unigenes were annotated with the protein databases of NR (NCBI, non-redundant), Swiss-Prot, KEGG (Kyoto Encyclopedia of Genes and Genomes), COG (Clusters of Orthologous Groups of proteins) and GO (Gene Ontology) as well as with the nucleotide database (NT). Unigene sequences are first aligned to protein databases like NR), Swiss-Prot, KEGG and COG (e-value<10e-5) by blastx, and to nucleotide database NT by blastn, retrieving proteins with the highest sequence similarity with the given unigenes along with their protein functional annotations.
(PDF)

S3 Table Identified peptides of *Chara* membrane proteins. (XLSX)

S4 Table Identified peptides of acidic and alkaline regions. (XLSX)

S5 Table Chara_australis_AllUnigene5-3_AA_only_longest (FASTA). (FA)

S1 Fig Characean thallus and cortical cytoplasm of an internodal cell. (A) Thallus of *Chara australis*. Internodes of the main axis and the branchlets are marked with black and red arrows, respectively. (B) pH-banding pattern in phenol red of an internodal cell. Pink colour indicates alkaline pH. (C) Schematic longitudinal section through an internodal cell showing the cortical cytoplasm with a charasome (red arrow), stationary chloroplasts (C), mitochondrium (M), peroxisome (P) and cortical endoplasmic reticulum (ER). A microtubule (MT) and an actin filament (AF) are seen at the plasma membrane (black line), subcortical actin filament bundles (AB) are located along the inner side of the chloroplasts. (D and E) FM1-43-labeled charasomes (green fluorescent) and mitotracker orange-stained mitochondria (red fluorescent) at an acid (D) and an alkaline band (E) from a light-exposed cell. (F) Electron micrograph of a cross-sectioned charasome labelled with an antibody against H^+^ ATPase. Labelling of the cell wall (CW) is unspecific. Bars are 1 cm (A), 300 μm (B), 10 μm (D and E), 500 nm (C and F). For methods see [3].
(TIF)

S2 Fig Distribution of unigene sequence lengths. Number of unigenes with lengths from 300 to 3,000 nucleotides (nt) is presented as log10 values.
(JPG)

S3 Fig Species distribution of the NCBI NR based *Chara* annotation. The percentage of all annotations of *Chara* unigenes to orthologous sequences of selected species is presented. The NCBI non-redundant (NR) database was searched.
(JPG)

S4 Fig Classification of the *Chara* transcriptome to COG categories. The identified *Chara* sequences were arranged into functional categories according to the COG (Clusters of Orthologous Genes) database.
(TIF)

S5 Fig Summary of GO classes. Identified transcripts of *Chara* cells are classified according to the GO (Gene Ontology) categories.
(TIF)

S6 Fig Distribution of *Chara* unigenes into MapMan BINs. Assembled unigenes were searched against TAIR (release 10), PPAP (Swiss-Prot Plant Protein), *Chlamydomonas* and *Physcomitrella* sequence databases with enabled InterProScan using Mercator software and classified into BIN classes (http://mapman.gabipd.org/mercator). Pies slices correlate with percentages of unigenes in the respective class. Number of BIN class and description is given in the legend with total unigenes numbers in brackets. Not assigned unigenes (ca. 60%) were omitted. The pie chart starts with ‚photosynthesis‘ at 12 o’clock with subsequent categories in counter-clockwise order (arrow).
(JPG)

S7 Fig Alignment of PM H^+^ ATPase sequences of *Chara* unigenes. *Chara* unigenes homologous to sequences of the PM H^+^ ATPase were translated into amino acid sequences and aligned with META7 software (MUSCLE algorithm). The *Chara* PM H^+^ ATPase CaHA1 was obtained from another sequencing project (access.-no. MF196972, Foissner & Hoepflinger, unpublished). The AHA2 protein of *Arabidopsis thaliana* (P19456.2) serves as a template to indicate specific domains: Transmembrane domains of AHA2 (Aramemnon, topology, AramTmMultiCon), peptide sequences identified in *Chara* membrane proteome, Phosphorylation site of phospho-intermediate state, C-terminus with Regulatory Domain I and Regulatory Domain II. Amino acids numbering is following the AHA2 sequence. Transmembrane domains of CaHA1 were determined by TMPred ([50]; (www.EXPASY.org)).
(PDF)

S8 Fig Whole original membranes/gels of Western blots. Lanes marked with red stars are shown in figures.
(PDF)

S9 Fig Phylogenetic analysis of PM H^+^ ATPase sequences. The evolutionary history was inferred by using the Maximum Likelihood method based on the JTT matrix-based model [71]. The bootstrap consensus tree inferred from 500 replicates [72] is taken to represent the evolutionary history of the taxa analysed [73]. Branches corresponding to partitions reproduced in less than 50% bootstrap replicates are collapsed. The percentages of replicate trees in which the associated taxa clustered together in the bootstrap test (500 replicates) are shown next to the branches [72]. Initial tree for the heuristic search were obtained automatically by applying Neighbour-Join and BioNJ algorithms to a matrix of pairwise distances estimated using a JTT model, and then selecting the topology with superior log likelihood value. The analysis involved 14 amino acid sequences. All positions containing gaps and missing data were eliminated. There were a total of 806 positions in the final dataset. Evolutionary analyses were conducted in MEGA7 [72].
(TIF)
