# Supplementary material for: Dissecting the subcellular membrane proteome reveals enrichment of H+ (co-)transporters and vesicle trafficking proteins in acidic zones of Chara internodal cells
Source: PLoS One. 2018 Aug 29;13(8):e0201480. doi: 10.1371/journal.pone.0201480 (PMC6114288; doi:10.1371/journal.pone.0201480)
Supplement: S1 Table — Sequence lengths are given in nucleotide numbers (nt) and base pairs (bp) or in percent for GC content, unknown nucleotides (N) or high quality reads (Q20). Q20 indicates the percentage of reads with an error rate ≤ 1%. N50 = 350 means that 50% of the reads could be assembled into contigs (or unigenes) with lengths > 350 bp. (PDF) [file pone.0201480.s001.pdf]

	total nucleotides (nt)	read numbers	GC content (%)	Q20 (%)	N content (%)	mean length (bp)	N50 (bp)
**Clean reads**	4,788,473,800	47,884,738	52.88	95.53	0	-	-
**Contigs**	66,986,851	-	-	-	-	253	350
**Unigenes**	49,842,575	-	-	-	-	619	878
