# Supplementary material for: Dissecting the subcellular membrane proteome reveals enrichment of H+ (co-)transporters and vesicle trafficking proteins in acidic zones of Chara internodal cells
Source: PLoS One. 2018 Aug 29;13(8):e0201480. doi: 10.1371/journal.pone.0201480 (PMC6114288; doi:10.1371/journal.pone.0201480)
Supplement: S2 Table — Unigenes were annotated with the protein databases of NR (NCBI, non-redundant), Swiss-Prot, KEGG (Kyoto Encyclopedia of Genes and Genomes), COG (Clusters of Orthologous Groups of proteins) and GO (Gene Ontology) as well as with the nucleotide database (NT). Unigene sequences are first aligned to protein databases like NR), Swiss-Prot, KEGG and COG (e-value<10e-5) by blastx, and to nucleotide database NT by blastn, retrieving proteins with the highest sequence similarity with the given unigenes along with their protein functional annotations. (PDF) [file pone.0201480.s002.pdf]

sample	NR	NT	Swiss-Prot	KEGG	COG	GO	all
all unigenes	28,450	16,087	18,630	19,681	14,978	16,580	35,118
