# Supplementary figures and images for: Dissecting the subcellular membrane proteome reveals enrichment of H+ (co-)transporters and vesicle trafficking proteins in acidic zones of Chara internodal cells
Source: PLoS One. 2018 Aug 29;13(8):e0201480. doi: 10.1371/journal.pone.0201480 (PMC6114288; doi:10.1371/journal.pone.0201480)

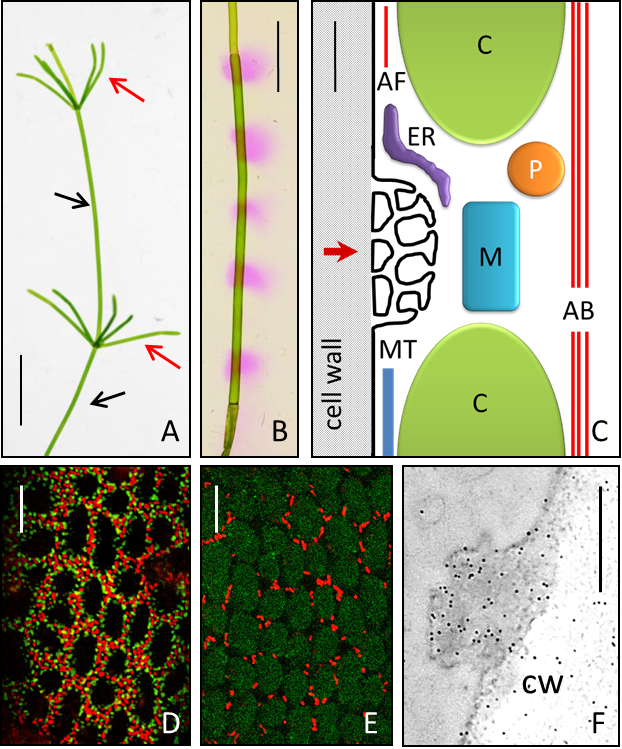

Supplement: S1 Fig — (A) Thallus of Chara australis. Internodes of the main axis and the branchlets are marked with black and red arrows, respectively. (B) pH-banding pattern in phenol red of an internodal cell. Pink colour indicates alkaline pH. (C) Schematic longitudinal section through an internodal cell showing the cortical cytoplasm with a charasome (red arrow), stationary chloroplasts (C), mitochondrium (M), peroxisome (P) and cortical endoplasmic reticulum (ER). A microtubule (MT) and an actin filament (AF) are seen at the plasma membrane (black line), subcortical actin filament bundles (AB) are located along the inner side of the chloroplasts. (D and E) FM1-43-labeled charasomes (green fluorescent) and mitotracker orange-stained mitochondria (red fluorescent) at an acid (D) and an alkaline band (E) from a light-exposed cell. (F) Electron micrograph of a cross-sectioned charasome labelled with an antibody against H+ ATPase. Labelling of the cell wall (CW) is unspecific. Bars are 1 cm (A), 300 μm (B), 10 μm (D and E), 500 nm (C and F). For methods see [3]. (TIF) [file pone.0201480.s006.tif]

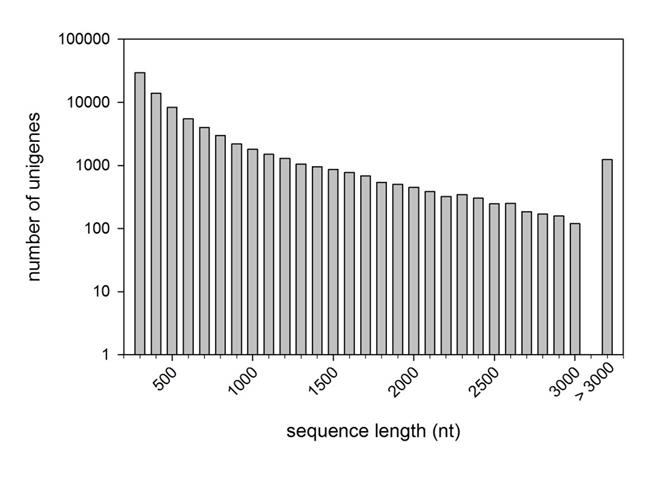

Supplement: S2 Fig — Number of unigenes with lengths from 300 to 3,000 nucleotides (nt) is presented as log10 values. (JPG) [file pone.0201480.s007.jpg]

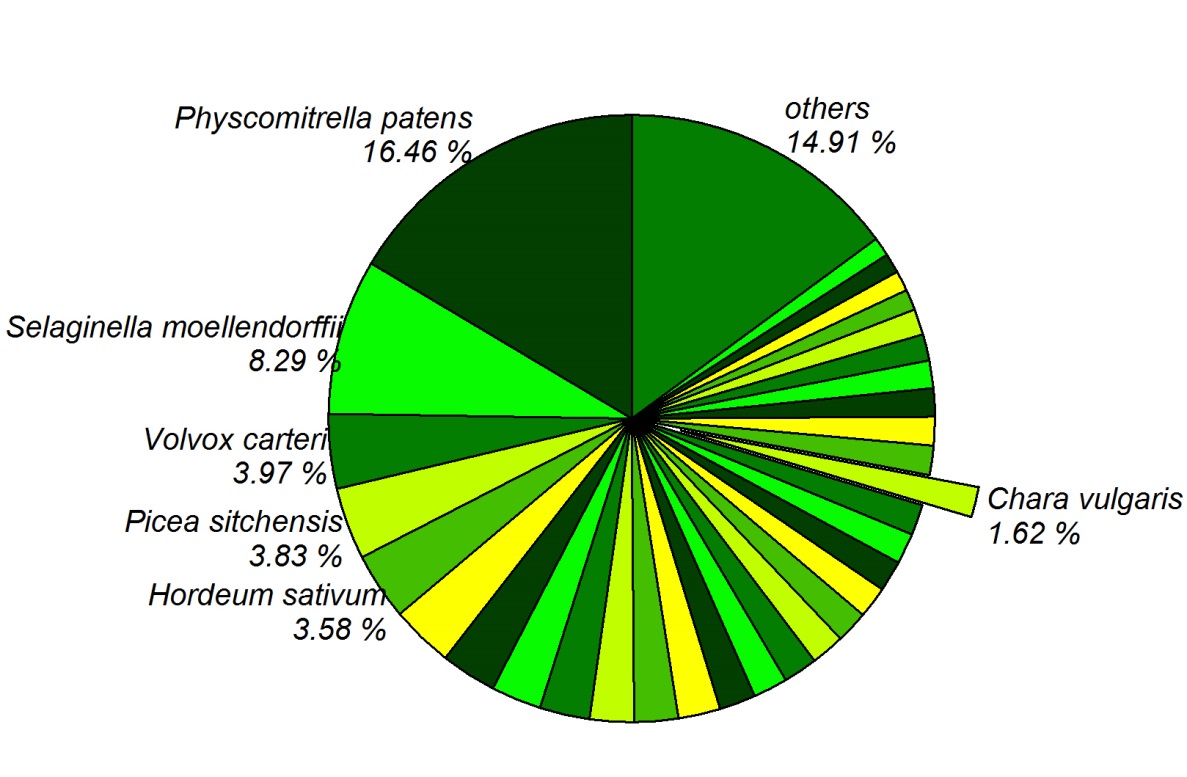

Supplement: S3 Fig — The percentage of all annotations of Chara unigenes to orthologous sequences of selected species is presented. The NCBI non-redundant (NR) database was searched. (JPG) [file pone.0201480.s008.jpg]

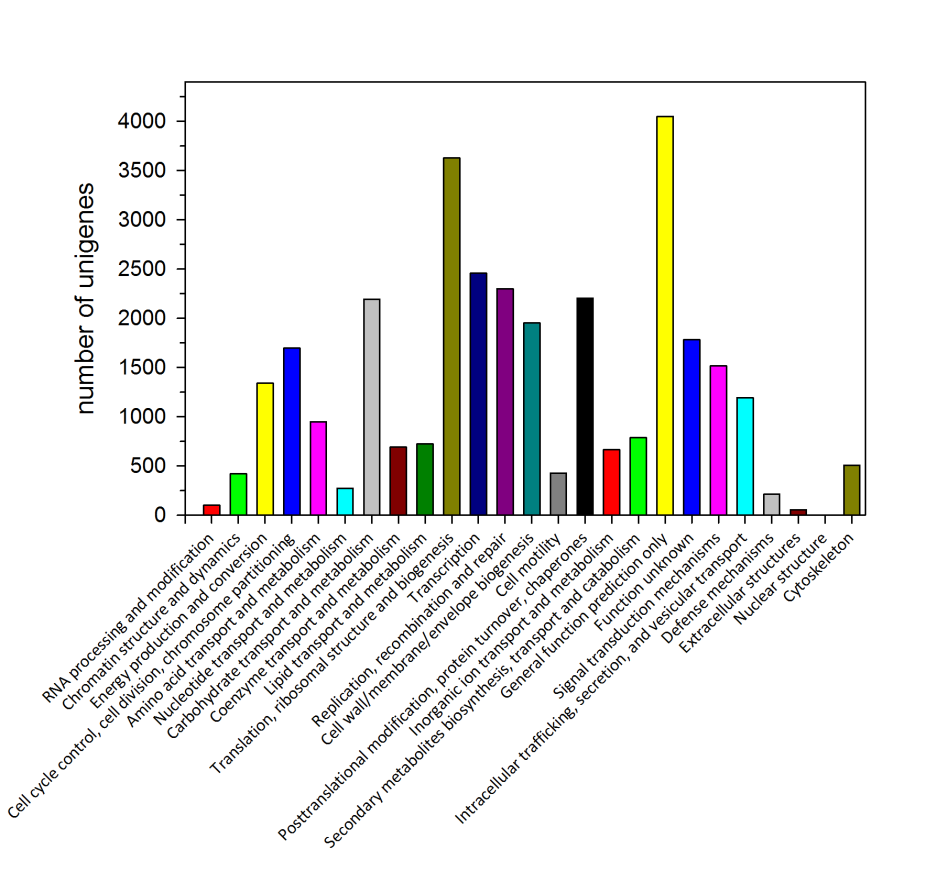

Supplement: S4 Fig — The identified Chara sequences were arranged into functional categories according to the COG (Clusters of Orthologous Genes) database. (TIF) [file pone.0201480.s009.tif]

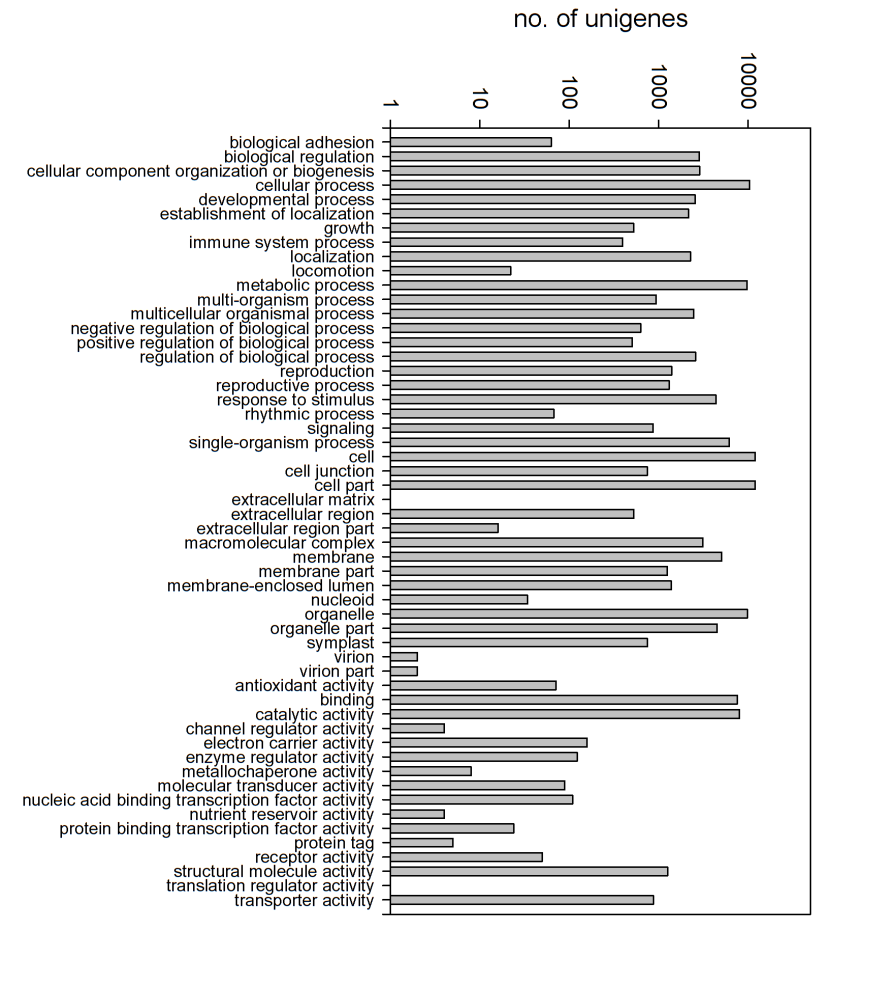

Supplement: S5 Fig — Identified transcripts of Chara cells are classified according to the GO (Gene Ontology) categories. (TIF) [file pone.0201480.s010.tif]

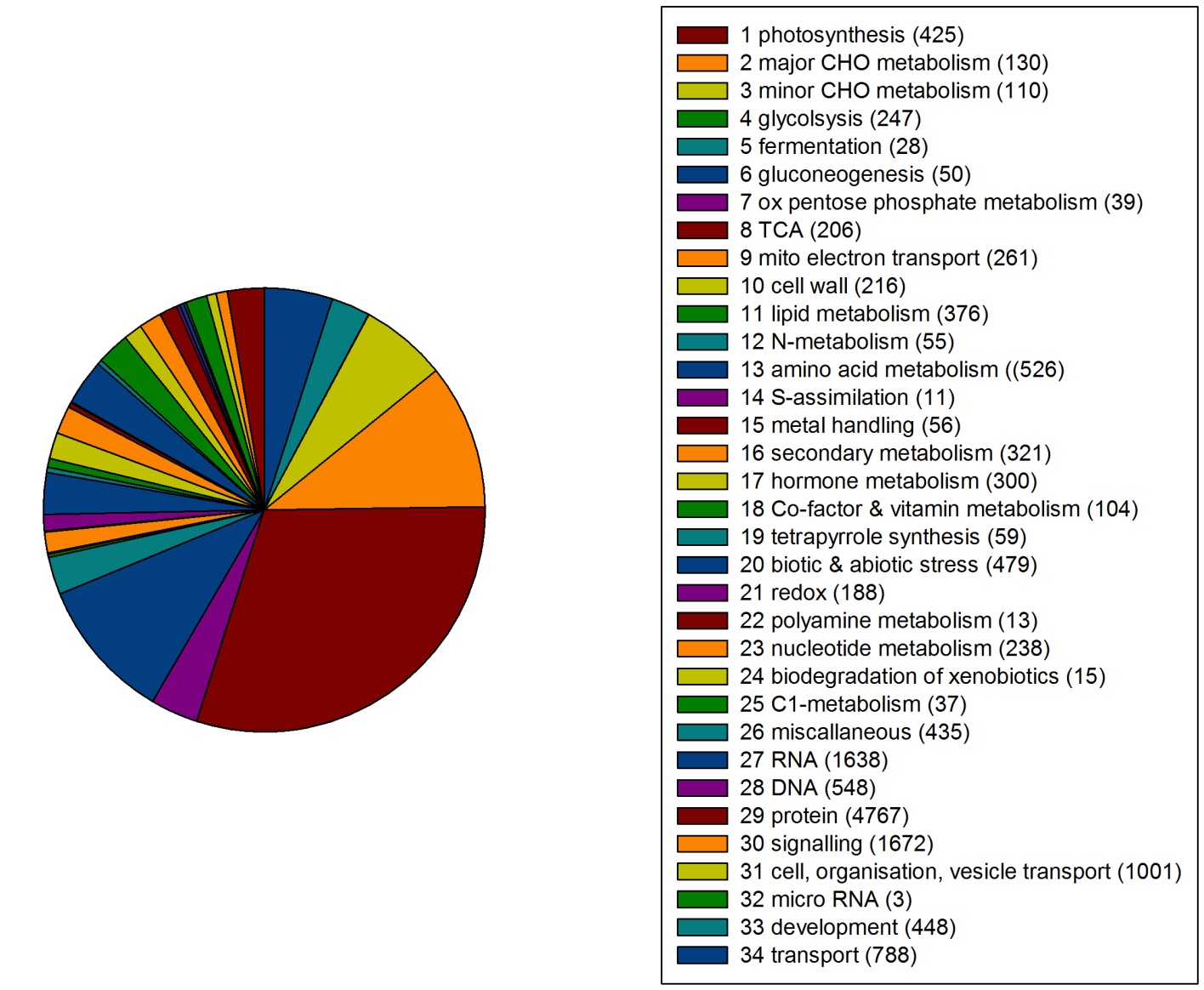

Supplement: S6 Fig — Assembled unigenes were searched against TAIR (release 10), PPAP (Swiss-Prot Plant Protein), Chlamydomonas and Physcomitrella sequence databases with enabled InterProScan using Mercator software and classified into BIN classes (http://mapman.gabipd.org/mercator). Pies slices correlate with percentages of unigenes in the respective class. Number of BIN class and description is given in the legend with total unigenes numbers in brackets. Not assigned unigenes (ca. 60%) were omitted. The pie chart starts with ‚photosynthesis‘ at 12 o’clock with subsequent categories in counter-clockwise order (arrow). (JPG) [file pone.0201480.s011.jpg]

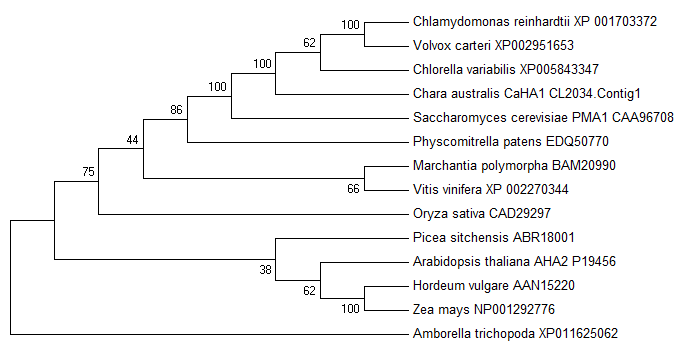

Supplement: S9 Fig — The evolutionary history was inferred by using the Maximum Likelihood method based on the JTT matrix-based model [71]. The bootstrap consensus tree inferred from 500 replicates [72] is taken to represent the evolutionary history of the taxa analysed [73]. Branches corresponding to partitions reproduced in less than 50% bootstrap replicates are collapsed. The percentages of replicate trees in which the associated taxa clustered together in the bootstrap test (500 replicates) are shown next to the branches [72]. Initial tree for the heuristic search were obtained automatically by applying Neighbour-Join and BioNJ algorithms to a matrix of pairwise distances estimated using a JTT model, and then selecting the topology with superior log likelihood value. The analysis involved 14 amino acid sequences. All positions containing gaps and missing data were eliminated. There were a total of 806 positions in the final dataset. Evolutionary analyses were conducted in MEGA7 [72]. (TIF) [file pone.0201480.s014.tif]
