# Supplementary material for: Dissecting the subcellular membrane proteome reveals enrichment of H+ (co-)transporters and vesicle trafficking proteins in acidic zones of Chara internodal cells
Source: PLoS One. 2018 Aug 29;13(8):e0201480. doi: 10.1371/journal.pone.0201480 (PMC6114288; doi:10.1371/journal.pone.0201480)
Supplement: S7 Fig — Chara unigenes homologous to sequences of the PM H+ ATPase were translated into amino acid sequences and aligned with META7 software (MUSCLE algorithm). The Chara PM H+ ATPase CaHA1 was obtained from another sequencing project (access.-no. MF196972, Foissner & Hoepflinger, unpublished). The AHA2 protein of Arabidopsis thaliana (P19456.2) serves as a template to indicate specific domains: Transmembrane domains of AHA2 (Aramemnon, topology, AramTmMultiCon), peptide sequences identified in Chara membrane proteome, Phosphorylation site of phospho-intermediate state, C-terminus with Regulatory Domain I and Regulatory Domain II. Amino acids numbering is following the AHA2 sequence. Transmembrane domains of CaHA1 were determined by TMPred ([50]; (www.EXPASY.org)). (PDF) [file pone.0201480.s012.pdf]

```
              1                       25                       50                       75                       100                      125
1  AHA2       MSSLEDIKNETVDLEKIPIEEVFQQLKCSREGLTTQEGEDRIQIFGPNKLEEKKESKLLKFLGFMWNPLSWVMEMAAIMAIALANGDGRPPDWQDFVGIICLLVINSTISFIEENNAGNAAAALMAGLAPKTK
2  CaHA1      -MGQDEGGKGGKHKNFVP-----------SKGLTSAEASNLLQIHGKNELEEKVTPSWVIFLRQLYAPMPILIWVAIIVEMAIKN-------WLDAAILLCIQLANATIGWYETTKAGNAVAALKASLKPRAT
3  CL2034.C1  -MGQDEGGKGGKHKNFVP-----------SKGLTSAEASNLLQIHGKNELEEKVTPSWVIFLRQLYAPMPILIWVAIIVEMAIKN-------WLDAAILLCIQLANATIGWYETTKAGNAVAALKASLKPRAT
4  UG10779    -MGLDEERKGGMPKDFVP-----------SKGLTSEEASNLLAIHGKNELEEKVTPSWLIFLRQLYAPMPILIWIAIIVELAIKN-------WLDAGILLIIQLCNATIGWYETTKAGNAVAALKASLKPRAT
5  CL4552.C1  -------------------------------------------------------------------------------------------------------------------------------------
6  UG30515    -------------------------------------------------------------------------------------------------------------------------------------
7  UG52361    -------------------------------------------------------------------------------------------------------------------------------------
8  UG55775    -------------------------------------------------------------------------------------------------------------------------------------
9  UG44920    -----------------P-DDFVF----SKEGLTSDEAAKRLEVYGRNQLPEHVDPKWLIFLRQFWAPMPIMIWIAIIIEVAIAN-------YIDMGILLFIQFANASISFYESTKAADAVAALK--------
10 UG45097    -------------------------------------------------------------------------------------------------------------------------------------

                   150                      175                      200                      225                      250                      275
1  VLRDGKWSEQEAAILVPGDIVSIKLGDIIPADARLLEGDPLKVDQSALTGESLPVTKHPGQEVFSGSTCKQGEIEAVVIATGVHTFFGKAAHLVDSTNQVGHFQKVLTAIGNFCICSIAIGMVIEIIVMYPIQRRKYRDGIDNLLVLL
2  VKRDGKVQTIDGSLLVPGDLVLLGAGSAIPADCIINEGT-IDVDTSALTGESMPETKYGGDEAQWGSTCVQGEVEATVTGTGKNTFFGRTAALLGDTNELGNIQKIILKITAGLVIISITLCAIALVYLLKGRNQDFIEALRFVVVLM
3  VKRDGKVQTIDGSLLVPGDLVLLGAGSAIPADCIINEGT-IDVDTSALTGESMPETKYGGDEAQWGSTCVQGEVEATVTGTGKNTFFGRTAALLGDTNELGNIQKIILKITAGLVIISITLCAIALVYLLKGRNQDFIEALRFVVVLM
4  VKRDGKVQTIDASVLVPGDLVLLGAGSAVPADCIINEGT-IDVDTSALTGESMPETKCGGDEAQWGSTCVQGEVEATVIGTGKNTFFGRTAALLTETNELGNIQKIILKITAGLVVISITLCAIALVYLLKGRDQDFIEALRFVVVLM
5  ----------------------------------------------------------------------------------------------------------------------------------------------------
6  ----------------------------------------------------------------------------------------------------------------------------------------------------
7  ----------------------------------------------------------------------------------------------------------------------------------------------------
8  ------------------------------------------VDQAALTGESLPVTFYKGDSCKMGSTVVRGETEGTVEFTGGNTFFGKTASLLVNTHEHTHIQ-IILMSIMFILVG-------------------------------
9  ----------------------------------------------------------------------------------------------------------------------------------------------------
10 ----------------------------------------------------------------------------------------------------------------------------------------------------

                     300                      325                      350                      375                      400                      425
1  IGGIPIAMPTVLSVTMAIGSHRLSQQGAITKRMTAIEEMAGMDVLCSDKTGTLTLNKLSVDKNLVEVFCKGVEKDQVLLFAAMASRVENQDAIDAAMVGMLADPKEARAGIREVHFLPFNPVDKRTALTYI-DGSGNWHRVSKGAPEQ
2  VASVPLAIEIVTTCTLAVGSRNLSAMNAIVTRLVSIEEMAGMNVLCSDKTGTLTLNKMVIQED-TPIFMRGETRETVIRSAALAAKWKEPPRDALDTMVLGTADLPSLDVYEQLDYMPFNPKVKRTEATLK-GPDGKVFKTTKGAPHI
3  VASVPLAIEIVTTCTLAVGSRNLSAMNAIVTRLVSIEEMAGMNVLCSDKTGTLTLNKMVIQED-TPIFMRGETRETVIRSAALAAKWKEPPRDALDTMVLGTADLPSLDVYEQLDYMPFNPKVKRTEATLK-GPDGKVFKTTKGAPHI
4  VASVPLAIEIVTTCTLAV----------------------------------------------------------------------------------------------------------------------------------
5  ----------------------------------------------------------------------------------------------------------------------------------------------------
6  -------------------------------------------VLCSDKTGTLTLNKMVIQDE-TPIFMRGESRDSVIRAAALAAKWKEPPKDALDTMVLGTADLHSLDVYQQLDYMPFNPKFKRTEATLR-SPDGKVFKTTKGAPHI
7  ----------------------------------------------------------------------------------------------------------------------------------------------------
8  ----------------------------------------------------------------------------------------------------------------------------------------------------
9  ----------------------------------------------------------------------------------------------------------------------------------------------------
10 ---------------------------------------------------------------------------SILMHAALAAKWKEPARDALDRLTLGSVDMSLLEDYEQLDFLPFDPTIKRTEGTVKHTKTGEVFKTTKGAPNI

                       450                      475                      500                      525                      550
1  ILE-LAKASNDLSKKVLSIIDKYAERGLRSLAVARQVVPEKTKESPGAPWEFVGLLPLFDPPRHDSAETIRRALNLGVNVKMITGDQLAIGKETGRRLGMGTNMYPSSALLGTHKDANLA---SIPVEELIEKADGFAGVFPEHKYEI
2  ILE-LCENKREIANEVNARVNQFGLRGIRCLAVAKS-------DE-GDRWRMMGVLTFLDPPRPDTKDTIERAQKFGVVVKMITGDQVVIALETARSLGLGTSIRGVQGLPSLGADNKIPKDLGKNYGRMILDCDGFAQVFPEHKYLI
3  ILE-LCENKREIANEVNARVNQFGLRGIRCLAVAKS-------DE-GDRWRMMGVLTFLDPPRPDTKDTIERAQKFGVVVKMITGDQVVIALETARSLGLGTSIRGVQGLPSLGADNKIPKDLGKNYGRMILDCDGFAQVFPEHKYLI
```

```
4  ----------------------------------------------------------------------------------------------------------------------------------------------------
5  ----------------------------------------------------------------------------------------------------------------------------------------------------
6  ILD-LCENKDEIANEVNARVNQFGLRGIRCLAVAKA-------DD-GQGWRMMGVLTFLDPPRPDTKDTIERAQKFGVVVKMITGDQVVIALETARSLGLGTSIRGVQGLPSLGEDNKVPKDLGKNYGPMILDCDGFA----------
7  ----------------------------------------------------------------------------------------------------------------------------------------------------
8  ----------------------------------------------------------------------------------------------------------------------------------------------------
9  ----------------------------------------------------------------------------------------------------------------------------------------------------
10 ILKLLGSSASDVHEAVENEVARLGAKGIRSLAVARG-------NEAGDEWKMLGLLTFLDPPRPDTKHTIDEAARMGVDVKMITGDHLLIAINTSAQLGMGQRIFTAERLPMLDEETKQ

       575                      600                      625                      650                                                         675
1  VKKLQERKHIVGMTGDGVNDAPALKKADIGIAVADATDAARGASDIVLTEPGLSVIISAVLTSRAIFQRMKNYTIYAVSITIRIVFGFMLIALIWE------------------------------------------FDFSAFMVLI
2  VEALRQIGCSVGMTGDGVNDAPALKRADVGIAVSGATDAARAASDIVLTSPGLSVVVEAIIVARCIFQRVKSFINYRIAATLQLLCFFFIAVFAFNPKDYQPPFCPGKGEPELYDSRWRHCIERHEKKKLEEDGWPEFFQLPVLLLML
3  VEALRQIGCSVGMTGDGVNDAPALKRADVGIAVSGATDAARAASDIVLTSPGLSVVVEAIIVARCIFQRVKSFINYRIAATLQLLCFFFIAVFAFNPKDYQPPFCPGKGEPELYDSRWRHCIERHEKKKLEEDGWPEFFQLPVLLLML
4  ----------------------------------------------------------------------------------------------------------------------------------------------------
5  NAALQLSDRMVGMTGDGVNDAPALKKAHVGIAVAGATEAAKGAADMILTKPGLSTIITAVTRSRKIFRRLESYVIYRLASSILVLLFTFFSIVALR------------------------------------------FQLPSWSLIL
6  ----------------------------------------------------------------------------------------------------------------------------------------------------
7  -------------------DAPALKRADIGIAVAGATDAARAAADIILTQEGLSTIIHGMEVAREIFQRISNFITYRISATLQLLFFFFIATFAFHPSDYEGPED-------------------------------------------

                        700                      725                      750                        775                      800
1  IAILNDGTIMTISKDRVKPSPTPDSWKLKEIFATGVVLGGYQAIMTVIFFWAAHKTDFFSDTFGVRSIRDNNH-ELMGAVYLQVSIISQALIFVTRSRSWSFVERPGALLMIAFLIAQLIATLIA--VYANWEFAKIRGIGWGWAGV-
2  ITLLNDGTLITIGYDYVIPSQNPEKWNLKVLGLASVVLAAVACFSSLLLLWAALDSWRTHSLFQRWGLRPIEYGQIVTMIYLKVSLSDFLTLFSSRTMGWFWTQRPGILLFCGALISLGISTIIAS-TFPNVEFEHMPIAGLAHAGAK
3  ITLLNDGTLITIGYDYVIPSQNPEKWNLKVLGLASVVLAAVACFSSLLLLWAALDSWRTHSLFQRWGLRPIEYGQIVTMIYLKVSLSDFLTLFSSRTMGWFWTQRPGILLFCGALISLGISTIIAS-TFPNVEFEHMPIAGLAHAGAK
4  ----------------------------------------------------------------------------------------------------------------------------------------------------
5  LSLVNDFTVMSTSLDNVYSSNNPLRWRMGYL

             825                      850                      875                      900                                       925
1  -------IWLYSIVTYFPLDVFKFAIRYILSGKAWLNLFENKTAFTMKKDYGKEEREAQWALAQRTLHGLQPKEAVNIFPEKGSYRELS-------------EIAEQAKRRAEIARLRELHTLKGHVESVVKLKGLDIETPSHYTV
2  YKMWTLWVWLYCILWWFI--QDGLKVAVYRLVVKYNVFGVNKGKEVFVKAPGKEVLDPVGNDNDVVTVSYSRASGKAMATQFKEGAAAATAQDTRLGREIGARYEVGEDGQEYLSVSYSREAGAAIAAELRAEMLKKSGATPPKYK
3  YKMWTLWVWLYCILWWFI--QDGLKVAVYRLVVKYNVFGVNKGKEVFVKAPGKEVLDPVGNDNDVVTVSYSRASGKAMATQFKEGAAAATAQDTRLGREIGARYEVGEDGQEYLSVSYSREAGAAIAAELRAEMLKKSGATPPKYK
```
