# Supplementary material for: Dissecting the subcellular membrane proteome reveals enrichment of H+ (co-)transporters and vesicle trafficking proteins in acidic zones of Chara internodal cells
Source: PLoS One. 2018 Aug 29;13(8):e0201480. doi: 10.1371/journal.pone.0201480 (PMC6114288; doi:10.1371/journal.pone.0201480)

Gel and membrane for Fig. 2A.

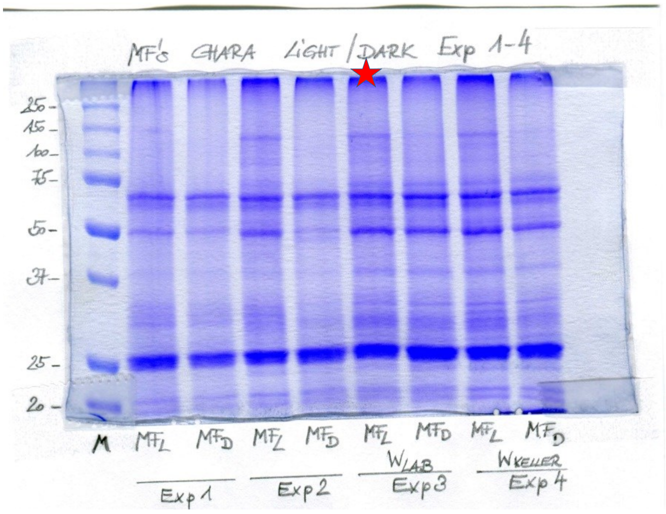

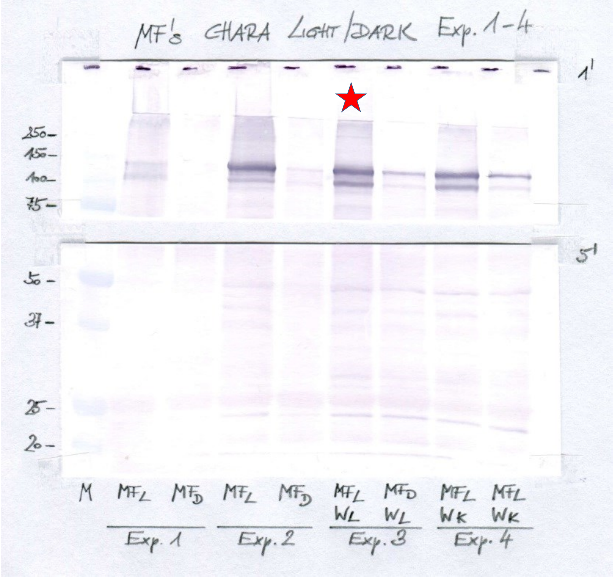

Anti PM H+ ATPase antibody

Anti actin antibody, not shown

Membrane for Fig. 5B

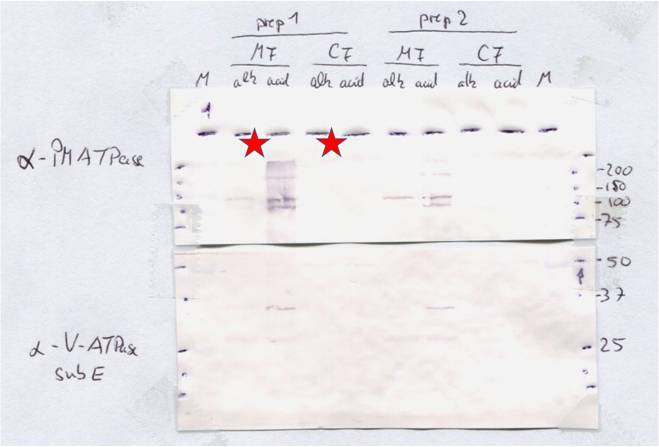

Membrane for Fig. 5C

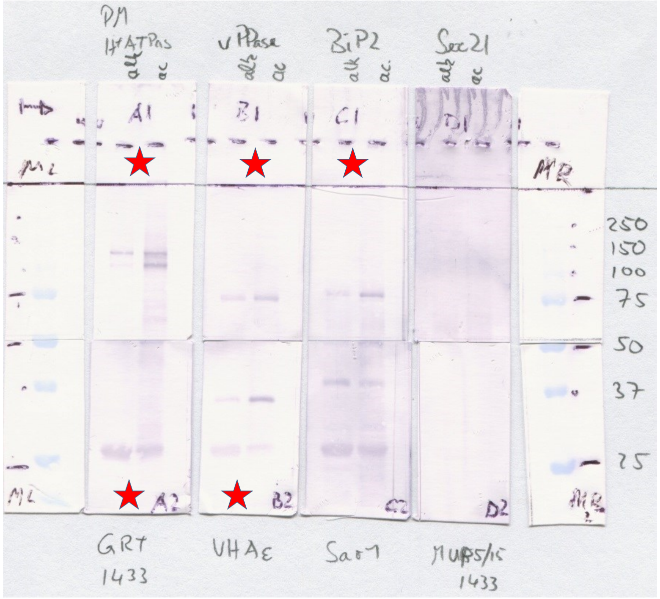

Supplement: S8 Fig — Lanes marked with red stars are shown in figures. (PDF) [file pone.0201480.s013.pdf]
